# The role of exercise in enhancing brain and cerebrovascular health via the bone–brain axis: implications for surgical and clinical interventions

**DOI:** 10.1097/JS9.0000000000003030

**Published:** 2025-07-17

**Authors:** Haojun Shi, Lei Huang, Qian Wang, Nan Zhang, Cui Lv, Chengshou Lin, Litao Shao, Dongshuai Xia, Zhijie Zhao, Guang Yang, Weijian Chen, Zhiwei Li, John H Zhang, Gongchang Yu, Liang Shi, Yisheng Chen

**Affiliations:** a Faculty of Chinese Medicine and State Key Laboratory of Quality Research in Chinese Medicines, Macau University of Science and Technology, Macau, China; b Fujian Key Laboratory of Toxicant and Drug Toxicology, Medical College, Ningde Municipal Hospital, Ningde Normal University, Ningde, Fujian, China; c Department of Central Laboratory, The Affiliated Taian City Central Hospital of Qingdao University, Taian, Shandong, China; d Science and Technology Innovation Center, Shandong First Medical University & Shandong Academy of Medical Science, Jinan City, Shandong Province, China; e School of Traditional Chinese Medicine, Shandong First Medical University & Shandong Academy of Medical Science, Jinan City, Shandong Province, China; f Department of Orthopaedics, Mindong Hospital Affiliated to Fujian Medical University, Fuan, Fujian, China; g Neck-Shoulder and Lumbocrural Pain Hospital of Shandong First Medical University, Shandong First Medical University & Shandong Academy of Medical Science, Jinan City, Shandong Province, China; h Department of Plastic and Reconstructive Surgery, Shanghai Ninth People’s Hospital, School of Medicine, Shanghai Jiao Tong University, Shanghai, China; i Department of Surgery of Spine and Spinal Cord, Henan Provincial People’s Hospital, People’s Hospital of Zhengzhou University, Zhengzhou, Henan, China; j Guangzhou University of Chinese Medicine, Guangzhou, Guangdong, China; k Clinical Laboratory Center, The People’s Hospital of Xinjiang Uygur Autonomous Region, Urumqi, Xinjiang, China; l Department of Neurosurgery, Department of Physiology and Pharmacology, Department of Neurosurgery and Anesthesiology, School of Medicine, Loma Linda University, Loma Linda, CA, USA; m Department of Molecular Cell and Cancer Biology, University of Massachusetts Medical School, Worcester, MA, USA; n Ningde Clinical Medical College, Ningde Normal University, Ningde, Fujian Province, China; o Department of Vascular surgery, Ningde Clinical Medical College, Fujian Medical University, Ningde, Fujian Province, China

**Keywords:** aging, bone–brain axis, cognitive function, exercise, inflammation, neurodegenerative diseases, neuroplasticity, osteocalcin, osteoporosis, therapeutic strategies

## Abstract

The bone–brain axis constitutes an interactive bidirectional signaling network integrating skeletal, neural, and muscular systems, serving critical functions across both normal physiological operations and disease mechanisms. Exercise, a non-pharmacological intervention, significantly influences this axis by enhancing bone-derived factors like osteocalcin, which in turn affect cognitive function, neuroplasticity, and systemic metabolism. This review delves into the molecular and cellular mechanisms underlying the bone–brain axis, with a particular focus on exercise-induced pathways. Physical activity stimulates the secretion of myogenic and neuromodulatory mediators including brain-derived neurotrophic factor (BDNF) and irisin, which augment synaptic adaptability, suppress inflammatory responses, and facilitate neuronal regeneration. These effects are critical in alleviating neurodegenerative diseases, osteoporosis, and mood disorders. The review also examines the endocrine role of bone-derived factors like uncarboxylated osteocalcin and sclerostin in modulating neurotransmitter synthesis and neuroimmune responses, including regulating osteoblast activity and bone remodeling, stimulating BDNF expression in the brain, regulating bone marrow-derived macrophages, and modulating cytokine pathways to alleviate neuroinflammation. Findings suggest that the bone–brain axis not only mitigates age-related degeneration but also holds therapeutic potential for chronic disease management. This review emphasizes the innovative application of exercise in optimizing bone–brain axis functionality, offering critical insights into its capacity to enhance cognitive performance, stabilize affective states, and promote systemic physiological resilience. By elucidating the intricate interplay between skeletal and neurobiological systems, this work paves the way for developing targeted interventions that can improve clinical outcomes in various surgical, orthopedic surgery and neurorehabilitation.

**Novel insights into the bone–brain axis: exercise-induced endocrine roles in cognitive and mental health:**

This review synthesizes emerging evidence linking skeletal system signaling to brain function, with a specific focus on the bone–brain axis as a mediator of exercise benefits. By integrating findings from molecular biology, neuroscience, and exercise physiology, we highlight novel endocrine roles of osteokines such as osteocalcin and irisin in promoting cognition and mental health. This interdisciplinary perspective contributes to the growing understanding of exercise as a systemic intervention for neurodegeneration.

**Learning points**:

- Exercise-induced bone signaling pathways, especially those involving osteocalcin and irisin, support cognitive function, neuroplasticity, and emotional regulation.
- The bone–brain axis represents a novel and promising therapeutic target for delaying or preventing neurodegenerative diseases.
- Creatine supplementation combined with exercise demonstrates synergistic potential in promoting both neurological and musculoskeletal health.
- Emerging interdisciplinary tools, including neuroimaging, artificial intelligence, and gene delivery systems, offer new avenues for personalized exercise-based interventions.

## Graphical abstract

**Figure:**
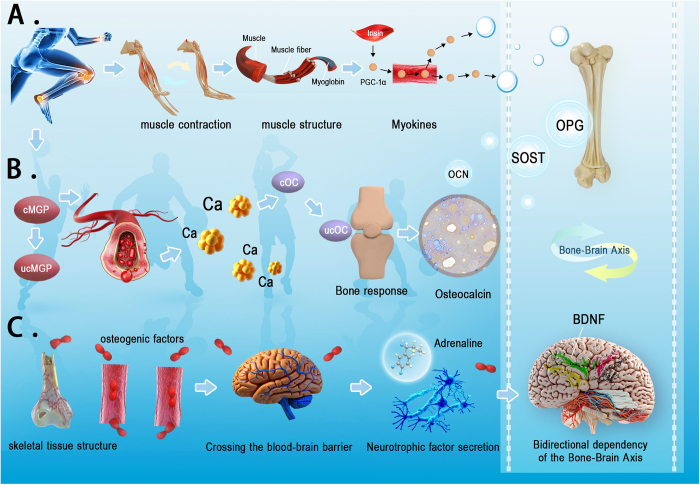

## Introduction

The human physiological network is complex and intricately interconnected, with the delicate interactions between the skeleton, muscles, brain, and nervous system constituting a key physiological mechanism known as the “bone–brain axis^[1,2]^.” Exercise plays an essential regulatory role in this axis[3]. The aging process induces progressive deterioration in cognitive capacity, skeletal integrity, and muscular mass among elderly populations[4]. These physiological declines substantially impair life quality while elevating risks of accidental falls and mortality[5]. Investigation of bone–brain axis mechanisms offers viable pathways for developing interventions to counteract such degenerative trajectories[6].

**Figure 1.**
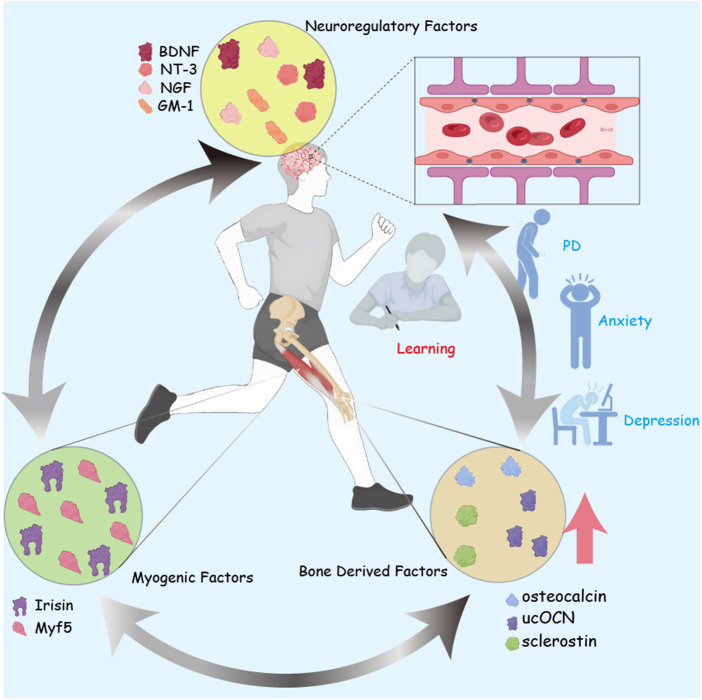
Mechanisms by which physical exercise promotes brain and cerebrovascular health via the bone–brain-muscle axis.

As a non-pharmacologic therapeutic approach, structured exercise demonstrates multidimensional health-promoting effects. Regular physical engagement enhances cardiocerebrovascular performance and metabolic regulation while mitigating chronic disease progression^[7,8]^. Exercise can be categorized into aerobic exercise and resistance training. Aerobic exercises, such as running and cycling, not only enhance cardiovascular function but also slow the degeneration of the hippocampus, while resistance training improves muscle strength, bone health, and reduces the risk of osteoporosis[9]. Given these benefits, exercise interventions have become a core component of the treatment for chronic diseases such as heart failure, depression, and multiple sclerosis, and even a first-line therapeutic option[10]. The impact of exercise on mental health has also become a research focus in recent years, involving its role in regulating mood, improving neurotransmitter imbalances, reducing stress hormone release, and promoting neurogenesis (Fig. 1)[11]. Studies have shown that regular physical activity can promote the synthesis of mood-regulating neurotransmitters such as 5-HT and BDNF, while lowering levels of stress hormones such as cortisol, thereby helping to alleviate symptoms of depression and anxiety[12]. The World Health Organization (WHO) identifies physical inactivity as the fourth primary contributor to global mortality, with 6% of annual deaths directly attributable to insufficient exercise. Prolonged sedentary behavior results in a 30% functional decline across tissues and organs, triggering cascading health complications[13]. During exercise, tissues such as muscles and bones secrete growth factors and cytokines that regulate the functions of the heart, brain, and skeletal muscles, promoting tissue remodeling and repair[14]. Age-related declines, such as reduced muscle strength, bone density, and brain function, are the result of multiple factors, including changes in neuromuscular function, protein synthesis, hormone levels, vascularization, and oxidative stress[15]. Research demonstrates that structured physical regimens, particularly resistance-based protocols, effectively optimize cognitive performance, musculoskeletal integrity, and age-related degeneration attenuation in older adults[16].

**HIGHLIGHTS**

- **Explores the bone–brain axis**: A bidirectional communication network linking the skeletal system, brain, and muscles, influencing brain health and cognitive function.
- **Role of exercise**: Examines how exercise enhances bone-derived factors like osteocalcin and irisin, which positively impact brain function, cognitive health, and neuroplasticity.
- **Impact on neurodegenerative diseases**: Highlights the potential of exercise in mitigating conditions like Alzheimer’s, Parkinson’s, and osteoporosis through the modulation of bone–brain interactions.
- **Molecular mechanisms**: Investigates the molecular and cellular pathways of exercise-induced changes in the bone–brain axis, including neurotransmitter regulation and neuroimmune responses.
- **Therapeutic potential**: Discusses exercise as a non-pharmacological intervention to optimize the bone–brain axis, offering a strategy for enhancing cognitive performance and mood regulation.
- **Focus on aging**: Reviews the benefits of exercise in elderly populations to prevent cognitive decline, enhance memory, and support bone health.

This figure illustrates the interconnected roles of neuroregulatory, myogenic, and bone-derived factors in mediating the positive effects of physical exercise on brain health and mental well-being. Physical activity stimulates the release of neuroregulatory factors, including brain-derived neurotrophic factor (BDNF), neurotrophin-3 (NT-3), nerve growth factor (NGF), and monosialoganglioside (GM-1). These factors enhance synaptic plasticity and cerebrovascular function, facilitating improved learning and memory. Exercise-induced myogenic factors, such as irisin and Myf5, contribute to neurogenesis and metabolic regulation, indirectly supporting brain health. Simultaneously, bone-derived factors, including osteocalcin, undercarboxylated osteocalcin (ucOCN), and sclerostin, modulate neuronal activity and cognition. This integrated regulation ameliorates symptoms of anxiety, depression, and neurodegenerative disorders such as Parkinson’s disease (PD). The figure highlights how exercise-driven molecular crosstalk between the skeletal, muscular, and nervous systems underpins systemic adaptations to enhance both brain function and mental health.

Amid escalating neurodegenerative disease burdens affecting aging demographics, cognitive deterioration emerges as a critical population health concern[17]. Beyond its structural role, skeletal endocrine functionality has gained substantial scientific interest, with contemporary research delineating reciprocal bone–brain communication networks termed the “bone–brain axis[18]This axis regulates brain function through the release of bone-derived cytokines and molecular signals, with some of these bone-derived factors able to cross the blood–brain barrier (BBB) and directly influence the health of the central nervous system[19]. The regulatory effects of exercise on the bone–brain axis have particularly drawn attention. Physical activity stimulates muscular release of signaling molecules including myostatin, critically sustaining physiological coordination between osseous and neurological systems[20]. For instance, the activation of exercise-induced transcription factors like PGC-1α not only improves the structure and function of bones and muscles but may also delay cognitive decline by influencing brain health[21]. Consequently, physical activity demonstrates considerable potential in chronic disease management and prevention as a non-drug therapeutic approach.

This review aims to systematically explore the mechanisms by which exercise promotes brain health through the bone–brain axis, particularly in the prevention of neurodegenerative diseases in the elderly and the enhancement of brain health. His analysis will systematically evaluate exercise’s geriatric impacts, elucidating its neuroregulatory pathways for depression mitigation by clarifying how physical activity modulates neurotransmitter and hormonal balance to reduce depressive manifestations and augment cognition. Special focus will address exercise’s therapeutic potential for neurobehavioral disorders including attention-deficit hyperactivity disorder (ADHD), examining the molecular pathways and biological substrates underlying symptom improvement through exercise interventions. Finally, this article will thoroughly discuss the multiple benefits of aerobic exercise for enhancing cognitive function, alleviating depression symptoms, and managing chronic pain, providing theoretical support for the development of more effective exercise intervention programs. This study complies with the TITAN Guidelines 2025[22].

## The biological basis of the bone–brain axis

### Bidirectional dependency of the bone–brain axis

In recent years, scientists have increasingly recognized that the relationship between the brain and the skeleton is not as unrelated as traditionally believed. Traditionally, the brain has been regarded as the “commander” of the body, while the skeleton is seen as the “supporter” and “protector.”[22] Emerging findings from advancing scientific investigations reveal an intricate mutual interdependency between cerebral and skeletal systems, as substantiated by contemporary research[23]. This bidirectional relationship is demonstrated by the brain’s regulation of bone health via neural pathways, while the skeleton influences brain function through endocrine signaling[24].

**Figure 2.**
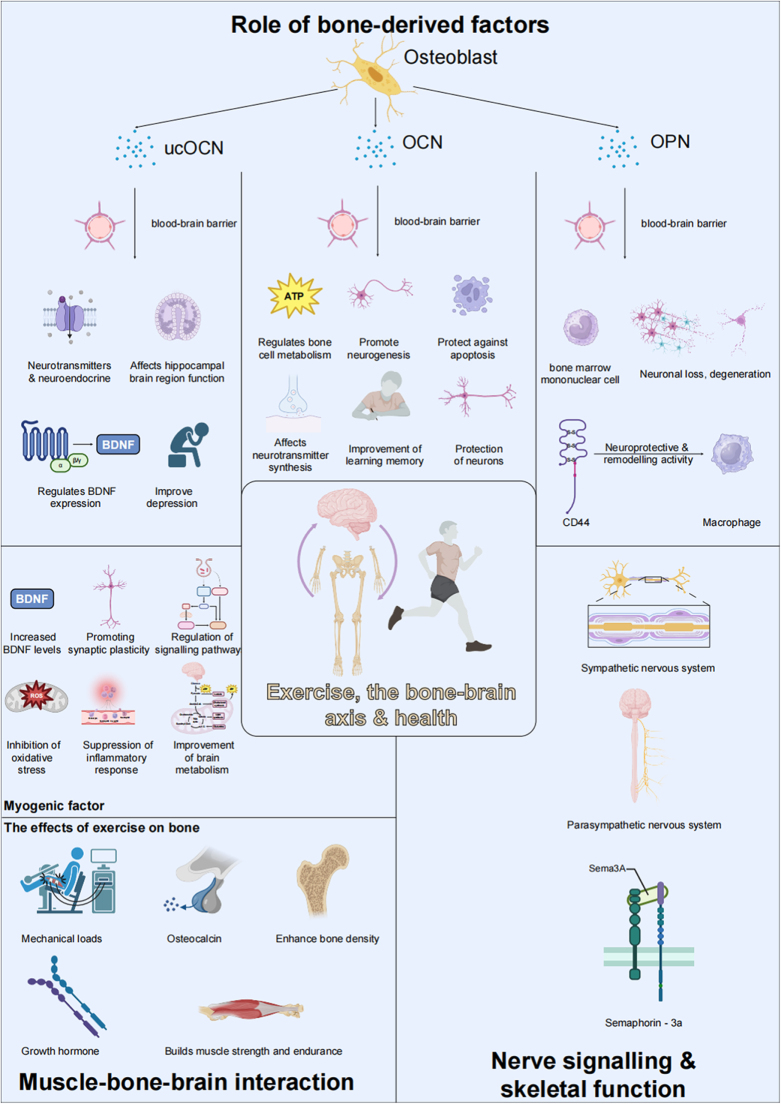
The role of bone-derived factors and their interactions with muscle and brain in the context of exercise and health.

First, the regulatory effect of the skeleton on the brain via “efferent nerves” has been extensively studied[25]. Osteoblast-derived uncarboxylated osteocalcin (ucOCN) traverses the BBB to modulate neurotransmitter production and neurotrophic factor expression, thereby affecting depression pathogenesis and therapeutic outcomes as referenced in Figure 2^[26,27]^. The cerebral mechanisms of ucOCN primarily involve monoaminergic neurotransmitter regulation (serotonin, norepinephrine, dopamine) and neuroendocrine pathway modulation (including BDNF expression), which collectively enhance cognitive capabilities, stimulate neural plasticity, and mediate exercise-induced skeletal-neural crosstalk[28]. ucOCN can specifically activate the Wnt/β-catenin signaling pathway in the hippocampus by binding to the Gprc6a receptor on the surface of neurons, thereby promoting synaptic plasticity and memory formation^[29,30]^. Single-cell RNA sequencing analysis showed that the pathway-related genes were differentially expressed in hippocampal neurons, and its biological significance was verified by PCA dimensionality reduction and functional enrichment analysis^[31,32]^. Exercise upregulates ucOCN expression in the skeleton, which in turn improves brain function by modulating the HPA axis, neuroimmune responses, and the expression of neurotrophic factors (Table 1)[28]. Simultaneously, the influence of the skeleton on the brain through “afferent nerves” has also garnered increasing attention[1]. Research confirms that sclerostin, a skeletal-secreted regulator, along with other osseous mediators, engages in bidirectional communication with the central nervous system via endocrine signaling mechanisms[33]. These factors not only participate in bone metabolism but also influence cognition, mood, and behavior through neural regulation[34]. For instance, irisin, a muscle-derived factor, promotes skeletal health and also regulates neurogenesis in the hippocampus, modulating neuroplasticity and improving brain function, particularly in the prevention and treatment of depression and cognitive dysfunction[35]. Irisin further strengthens the bidirectional dependency of the bone–brain axis by regulating G protein-coupled receptors and other targets. GPR20 Linked to irisin-mediated neuroprotection and BDNF upregulation in the hippocampus. GPR56 regulates osteoblast differentiation and synaptic plasticity through RhoA/ROCK signaling. These receptors mediate irisin’s dual effects on bone formation (via Wnt/β-catenin activation) and brain function (via TrkB/BDNF pathways)[36].

This figure illustrates the critical roles of bone-derived factors, including osteocalcin (OCN), undercarboxylated osteocalcin (ucOCN), and osteopontin (OPN), in mediating brain and skeletal health through the bone–brain axis. These factors, secreted by osteoblasts, cross the BBB to influence various neurophysiological processes. ucOCN regulates neurotransmitter synthesis, enhances hippocampal brain function, and modulates brain-derived neurotrophic factor (BDNF) expression, contributing to the alleviation of depression. OCN promotes neurogenesis, protects neurons from apoptosis, and improves learning and memory by regulating cellular metabolism in the brain. OPN plays a role in reducing neuronal degeneration, exhibiting neuroprotective and remodeling activities, and interacting with immune cells such as macrophages via CD44 signaling.The figure also emphasizes the impact of exercise on the bone-muscle-brain axis. Mechanical loads and exercise-induced factors enhance bone density, promote muscle strength and endurance, and improve brain metabolism by reducing oxidative stress and inflammatory responses. These processes contribute to synaptic plasticity and improved neural signaling. Furthermore, the involvement of the sympathetic and parasympathetic nervous systems in skeletal regulation through pathways such as Semaphorin-3A signaling highlights the complex interplay between these systems in maintaining systemic health. This visual representation underscores the central role of exercise in modulating the bone–brain-muscle network and promoting overall physiological well-being.

**Table 1:** Biological basis of the bone–brain axis

	Name	Source	Properties	Mechanism	Example
Effects of bone-derived factor on the brain	Osteocalcin	Osteoblasts	Non-collagen	Regulates the expression of neurotransmitters and neurotrophic factors in the brain, affecting emotions, cognition and behavior.	-
	Sclerostin	Osteocytes	Secretory glycoprotein	Regulates neuronal growth and repair through interaction with neurotransmitters	-
Effects of neuromodulation on bones	Neurotransmitters	Nervous system	Amino acids, peptides, etc.	Binds to receptors on neurons and bone cells in the bone marrow to regulate bone formation	Norepinephrine, epinephrine, glutamate
	Neurohormones	Neuroendocrine cells	Mostly peptides	Regulates osteoblasts, osteoclasts and bone marrow stem cells, regulating bone formation	Adrenal cortex hormones, oxytocin, prolactin
	Sympathetic and parasympathetic nerves	-	-	Regulates bone health and metabolism through neurotransmitter release, nerve–bone interactions, and bone biological responses	-
	Neuroinflammation	Nervous system	-	Influencing bone health through neurotransmitters and immune factors	Cytokines, chemokines, etc.

Neural modulation’s critical role in osseous homeostasis is gaining scientific recognition. The sympathetic nervous system (SNS) modulates bone remodeling and mineral equilibrium through specialized regulatory mechanisms[37]. Scientific investigations confirm direct correlations between sympathetic tone and osseous mass/density metrics[38]. Hyperactivation of sympathetic signaling may suppress osteogenic processes, whereas balanced neural input supports skeletal integrity[39]. Concurrently, parasympathetic pathways demonstrate regulatory significance in osseous metabolism, particularly through coordinating bone resorption-formation dynamics, reinforcing the nervous system’s multifaceted contributions within the bone–brain axis framework[40]. These findings indicate that the bone–brain axis is not a unidirectional chain of influence, but rather a dynamic, bidirectional feedback system where the skeleton and the brain are interdependent and mutually regulating[2]. The brain modulates bone metabolism through neural pathways, while the skeleton impacts brain health through endocrine factors and feedback signaling^[24,41]^. This reciprocal regulatory framework enhances comprehension of cerebral-skeletal crosstalk while revealing novel treatment perspectives for addressing neurodegenerative pathologies, affective disorders, and related clinical manifestations.

### Role of bone-derived factors in the bone–brain axis

Skeletal tissue secretes diverse bioactive mediators that have gained substantial research focus for their functional contributions within the bone–brain axis framework[42]. The skeleton is not just a structural support for the body, it also regulates brain function and emotion through the secretion of specific molecules[43]. Bone-derived factors such as OCN, ucOCN, and sclerostin (SOST) play crucial roles in bone metabolism and also exert profound effects on other organs and systems, including the brain (Fig. 2)^[33,44]^. In patients with Alzheimer’s disease (AD), the decrease of osteocalcin level is significantly negatively correlated with β starch-like protein deposition and Tau protein phosphorylation, suggesting that bone-derived factors may delay disease progression by inhibiting neuroinflammation and oxidative stress[45] .Bone-derived factors exert their effects through distinct molecular pathways that bridge skeletal and neurological systems.

#### Interaction between osteocalcin and the brain

Produced by osteoblasts, the non-collagenous protein osteocalcin demonstrates pivotal involvement in skeletal metabolic regulation, as extensively documented in bone physiology research[46]. More importantly, the ucOCN can enter the bloodstream and cross the BBB to influence neural activity (Table 1)^[26,47]^. Research has shown that ucOCN regulates the expression of neurotransmitters and neurotrophic factors in the brain, thereby affecting mood, cognition, and behavior[28]. Particularly, ucOCN plays a pivotal role in the onset of depression and cognitive impairment. It enhances the synthesis of monoamine neurotransmitters such as serotonin (5-HT) and norepinephrine, as well as the expression of BDNF, which improves depressive symptoms, promotes neuroplasticity, and enhances memory function^[48,49]^. In the brain, ucOCN binds to GABAergic neurons in the ventral tegmental area (VTA) and raphe nuclei, directly upregulating tryptophan hydroxylase 2 (TPH2) to enhance serotonin synthesis. Concurrently, it inhibits synaptic GABA transporters (GATs), prolonging serotonin availability. Moreover, the effects of exercise on osteocalcin are of significant importance. Exercise upregulates osteocalcin expression, thereby regulating neurotrophic factors in the brain and exerting antidepressant, anxiolytic, and cognitive-enhancing effects[50]. Studies have found that exercise, by modulating ucOCN secretion, regulates the HPA axis, inflammatory responses, and the neuroendocrine system, thus helping to counteract mood disorders and neurodegenerative diseases[28].

#### Sclerostin and its relationship with the brain

Sclerostin, an inhibitory modulator secreted by osteocytes, plays a principal role in skeletal remodeling processes[51]. While its skeletal regulatory functions are scientifically validated, emerging investigations focus on its neurobiological implications. Current evidence indicates sclerostin’s dual functionality in maintaining osseous homeostasis while potentially mediating central nervous system activities through endocrine signaling routes[52]. Some research has found that sclerostin, by acting on specific receptors in the bone marrow, may modulate brain function through neuroimmune mechanisms, thereby influencing mood and cognitive ability[53]. The effects of sclerostin on the brain might be related to its role in bone metabolism. It is believed that sclerostin, in conjunction with osteocalcin, helps regulate the balance of the bone–brain axis[54]. The study of gene expression and regulation mechanism reveals that this regulation may occur through the interaction with neurotransmitters, which regulates the growth and repair of neurons. Sclerostin may interact with the neuroimmune pathway in two key ways, including Wnt/β-catenin-dependent inhibition of microglia activation and astrocyte-mediated NF-κB/IL-6 signaling. Reduced microgliosis and TNF-α levels correlate with improved cognitive function in the SOST gene knockout model, highlighting its role in the regulation of neuroinflammation[55].Emerging investigations highlight sclerostin’s potential as a novel therapeutic candidate, particularly for addressing neurodegenerative pathologies and affective disorders, demonstrating significant clinical translation prospects.

#### Bone-derived factors and their relationship with neuroinflammation

In addition to regulating neurotransmitters, bone-derived factors also influence neuroinflammation by modulating neuroimmune responses[6]. Factors like osteocalcin can cross the BBB and regulate the activity of neuroimmune cells, thereby playing a role in modulating the brain’s immune environment^[2,26]^. For example, research has shown that ucOCN can suppress the secretion of inflammatory cytokines by regulating T-cell and B-cell responses, thus reducing neuroinflammatory reactions[56]. This discovery elucidates novel regulatory dimensions of osseous signaling molecules in neuroimmune modulation, unveiling potential therapeutic targets for anti-neuroinflammatory pharmaceutical development. The bone–brain axis exhibits multidimensional functional integration through skeletal-derived mediators, demonstrating complex bidirectional regulatory capacities. They affect brain function by modulating neurotransmitters, neurotrophic factors, and neuroimmune responses^[41,42]^. In the future, bone-derived factors will be studied in combination with bioinformatics technology, which is expected to reveal their potential application in the treatment of neurodegenerative diseases, emotional disorders, cognitive disorders and other diseases, and provide a new strategy for clinical intervention and prevention of bone–brain axis^[57,58]^^(p610581)^. Recent advances in single-cell RNA sequencing have further elucidated the heterogeneity of neuroimmune cell populations influenced by bone-derived factors, providing deeper insights into their roles in neuroinflammation and potential therapeutic targets^[59,60]^.

### Mechanisms of muscle–bone–brain interactions

The interactions between muscles, bones, and the brain are mediated by complex biological pathways, forming a multi-level physiological network[61]. Contemporary research has revealed that muscular systems exert regulatory influences beyond osseous metabolism, modulating skeletal and neurological activities via myokine-mediated signaling pathways[62]. The interactions among muscles, bones, and the brain are not confined to providing physical structural support but are primarily connected via endocrine, neural, and immune pathways, influencing overall health[63].

#### Role of muscle-derived factors

Studies have shown that the extracellular matrix (ECM) significantly affects stem cell differentiation[64]. Muscles secrete a variety of factors, such as irisin, muscle-derived growth factor (MGF), and other cytokines, which regulate the functions of bones and the brain (Table 1)[62]. The method of single-cell transcriptome analysis refers to a number of studies, including data processing, cell subpopulation identification and pathway enrichment analysis, and reveals that the differences of these factors in the musculoskeletal-brain axis reach^[65,66]^. Irisin, a hormone secreted after muscle contraction, circulates through the bloodstream, promoting bone formation and enhancing neuroplasticity[67]. The main effect of irisin is its activation of neurogenesis in the hippocampus, thereby improving learning and memory[68]. Additionally, irisin exerts antidepressant and anxiolytic effects by improving brain blood flow, reducing inflammation, and promoting neuronal growth[69]. Furthermore, the influence of MGF on bones should not be overlooked. Basic research shows that the regulatory mechanism of specific genes on mesenchymal stem cell differentiation provides a theoretical basis for cell therapy and further explains the action pathway of muscle-derived factors. MGF not only promotes increased bone density but also regulates the balance between bone formation and resorption, impacting bone health[70]. MGF binds to IGF-1R with high affinity, triggering autophosphorylation of the receptor’s tyrosine kinase domain. MGF activates PI3K/Akt signaling, promoting osteoblast survival and differentiation via mTORC1. In addition, MGF upregulates BDNF expression in neurons via IGF-1R/TrkB co-activation, enhancing synaptic plasticity. These factors are involved not only in the dynamic regulation of bones but also in modulating mood, cognition, and neuroregeneration through their effects on the brain. Emerging evidence highlights Melilotus officinalis (L.) Pall. as a potential adjunct to exercise, modulating inflammatory pathways (e.g., NF-κB/IL-10) and promoting growth factor secretion (e.g., IGF-1) to enhance muscle–bone–brain crosstalk^[71,72]^.

#### Neuro-muscular-bone feedback mechanisms

There exists a bidirectional regulatory mechanism between muscles, bones, and the brain, which enhances the flexibility and adaptability of the system^[73,74]^. For example, during physical activity, muscles release factors like irisin, which act on bones to enhance bone density and quality[75]. Concurrently, these bioactive mediators traverse the BBB to access cerebral regions, stimulating neural regeneration and repair mechanisms[69]. Neurological activity reciprocally modulates musculoskeletal homeostasis. Physical exercise exhibits intrinsic neuroprotective correlations, particularly through cognitive enhancement, depressive symptom mitigation, and emotional regulation optimization[76]. By regulating muscle and bone function, exercise promotes the formation and maintenance of neural networks[77]. Moreover, the nervous system perceives and regulates the bone remodeling process through movement[78]. Neural signaling pathways modulate osteoblast/osteoclast dynamics within bone marrow microenvironments, thereby regulating skeletal architecture and biomechanical functionality^[79,80]^. The motor cortex in the brain plays a crucial role in this interaction, as it controls the signals that regulate muscle movements, which further affect bone load and structure, transmitting neural signals to bone cells to promote bone renewal[73].

#### Interaction of neural-immune pathways

The mechanisms of muscle–bone–brain interactions also involve the intertwining of neural-immune pathways[81]. The factors secreted by muscles not only regulate bone and brain function but also influence the health of the nervous system by modulating neuroimmune responses^[62,82]^. Scientific investigations confirm that myokines including irisin mitigate cerebral inflammation, consequently reducing neuronal degeneration while enhancing affective states and cognitive performance[69]. Concurrently, osteoblast-derived cytokines modulate cerebral immune responses, exerting substantial regulatory effects on neural circuitry and emotional processing[83]. The neuroimmune system’s regulatory scope extends beyond mood and cognition modulation to encompass neurodegenerative pathology mechanisms[84]. Activated neuroimmune pathways demonstrate strong correlations with AD and PD pathogenesis[85]. Musculoskeletal interventions through neuroimmune regulation may provide critical neuroprotective benefits against disease initiation and progression.

#### Exercise and muscle–bone–brain interactions

Exercise, as a powerful external stimulus, can produce significant physiological effects by regulating the interactions between muscles, bones, and the brain[73]. Physical activity enhances musculoskeletal integrity while optimizing osseous density and structural competence, simultaneously modulating bone–brain axis functionality[86]. More importantly, exercise activates the nervous system, regulating mood, cognitive function, and neurogenesis[87]. Physical training stimulates myokine release, augmenting osseous tissue formation and neural adaptability to mitigate psychological disorders including depressive and anxiety symptoms[11]. Additionally, exercise increases blood circulation, promoting nutrient supply and waste clearance for neurons, thereby maintaining brain health[88]. In this way, the health of muscles and bones has a positive regulatory effect on brain function, forming a complete physiological regulation system[89].

### Neural regulation of bone

The nervous system not only regulates movement, sensation, and cognition but also influences bone physiological functions through the neural–bone axis. Recent research indicates that the neural regulation of bones is achieved through multiple mechanisms involving neurotransmitters, neurohormones, and physiological responses related to the nervous system[24]. Neuromodulatory mechanisms exert critical regulatory influences on osseous tissue formation, structural adaptation, and metabolic homeostasis, while simultaneously determining key parameters including mineral density, microarchitectural integrity, and biomechanical competence.

#### Impact of neurotransmitters on bone

The nervous system directly or indirectly regulates bone metabolism through the release of neurotransmitters[90]. Methodology based on multi-omics integration combined with big data analysis comprehensively reveals the complex network of neurotransmitters and bone metabolism[91].For example, the roles of norepinephrine, epinephrine, and other neurotransmitters in bone metabolism have gained widespread attention[92]. Through β-adrenergic receptor binding in osseous tissues, norepinephrine stimulates osteoblast growth and specialization while suppressing osteoclast functionality, thereby maintaining skeletal homeostasis and metabolic equilibrium[93]. Molecular analyses have confirmed gene expression alterations, reinforcing mechanistic insights into neurotransmitter-mediated bone metabolic regulation[94]. As a key modulator of osseous metabolism, norepinephrine critically influences skeletal disorder pathogenesis, including osteoporosis development[92]. Neurotransmitters such as glutamate and γ-aminobutyric acid (GABA) further modulate osseous functionality via neuro-osseous signaling pathways[95]. Glutamate, as a major excitatory neurotransmitter, promotes bone formation by binding to receptors on neurons and bone cells in the bone marrow[96]. Functioning as the primary inhibitory neural transmitter, GABA suppresses osseous tissue degradation through curtailing osteoclastic resorptive processes, thereby preserving skeletal homeostasis.

#### Impact of neurohormones on bone

Neurohormones including adrenal corticosteroids, oxytocin, and prolactin exert critical regulatory functions in bone-related neural signaling[97]. Research demonstrates oxytocin’s substantial regulatory effects on osseous metabolism, particularly during bone repair and regeneration phases. This neuropeptide activates osteoblastic activity and bone marrow-derived stem cells to accelerate bone formation[98]. Furthermore, oxytocin stabilizes skeletal microarchitecture and mitigates bone loss by mediating neuro-osseous metabolic crosstalk[97]. Conversely, prolonged corticosteroid administration compromises osseous health by suppressing osteoblast differentiation while enhancing osteoclastic resorption, ultimately reducing mineral density and elevating fracture susceptibility[99]. However, over short periods, adrenal corticosteroids may help maintain bone function and stability during stress responses[100].

#### Regulation of bone by sympathetic and parasympathetic nervous systems

The autonomic nervous system demonstrates dual regulatory control over skeletal homeostasis, with sympathetic pathways mediating norepinephrine-driven bone resorption and remodeling processes[101]., typically correlating with diminished mineral density and elevated fracture risks[39]. Conversely, parasympathetic activation counterbalances these effects by enhancing osteogenic activity and maintaining structural integrity[102]. The interaction between the sympathetic and parasympathetic nervous systems regulates bone health and metabolism through the release of neurotransmitters, neural–brain interactions, and biological responses of bones[40]. This bidirectional regulation of bones by the nervous system provides dynamic balance for bone stability and offers new approaches for preventing and treating related diseases.

#### Impact of neuroinflammation on bone metabolism

Significant bidirectional interactions exist between neural inflammatory responses and osseous metabolism[103]. Scientific investigations confirm that central nervous system inflammation modulates skeletal health through neuromodulators and immunoregulatory mediators[41]. These neuroinflammatory processes demonstrate strong pathological correlations with osteoporosis, osteoarthritis progression, and impaired fracture healing mechanisms[104]. Neural-derived inflammatory mediators, including cytokine and chemokine cascades, alter bone marrow immune cell dynamics, consequently modifying bone remodeling equilibrium[79]. For instance, neural inflammation stimulates osteoclastic resorption via pro-inflammatory agents like tumor necrosis factor (TNF) and interleukin (IL) secretion[105], while concurrently suppressing osteoblastic proliferation and differentiation to impede skeletal regeneration[106].

#### Neural regulation in osteoporosis

Neural modulation serves as a critical determinant in osseous metabolic pathologies including osteoporosis[24]. Scientific evidence establishes pathophysiological connections between osteoporosis pathogenesis and neural dysregulation, particularly sympathetic hyperactivation that correlates with accelerated bone mass depletion and elevated fracture risks (Table 1)[107]. Therapeutic innovation targeting neural pathways shows promise for osteoporosis management, exemplified by pharmacological suppression of excessive sympathetic signaling or neurohormonal modulation of osseous metabolism to ameliorate clinical manifestations and reduce fracture incidence^[57,108]^.

### The relationship between the bone–brain axis and diseases

Emerging research highlights the skeletal-neural signaling network, a sophisticated bidirectional communication pathway, as a critical focus in contemporary biomedical studies^[41,42]^. Scientific evidence confirms this cross-system interaction serves as a critical determinant in the pathogenesis of neurodegenerative disorders, osteoporosis spectrum conditions, affective disorders, and AD pathology[18]. Pathological alterations in this regulatory framework are implicated in chronic disease etiology, particularly within geriatric cohorts where axis dysfunction correlates with disease manifestation[2].

#### Relationship between osteoporosis and neurodegenerative diseases

Osteoporosis manifests as reduced mineral density and heightened skeletal fragility resulting from imbalanced bone remodeling processes (excessive resorption/inadequate formation)[109]. Age-related pathophysiological correlations reveal frequent comorbidity and reciprocal modulation between osteoporotic conditions and neurodegenerative pathologies including AD and PD[110]. The bone–brain axis has been scientifically confirmed to exert bifunctional regulatory effects on both osseous integrity and neural homeostasis[42]. A decline in bone quality may directly or indirectly affect brain health, and conversely, brain dysfunction may also impact bone metabolism[111]. Osteoporosis patients frequently exhibit cognitive decline, especially among the elderly[112]. Experimental studies suggest that bone-derived factors such as osteocalcin, ucOCN, and SOST may influence neuronal activity and cognitive function through the bone–brain axis mechanism[42]. Furthermore, the interaction between the skeletal and nervous systems may lead to neurodegenerative changes through alterations in neurotransmission, neuroinflammation, and other pathways[113].

#### Relationship between depression and the bone–brain axis

Depression is another disease closely linked to the bone–brain axis. Clinical observations indicate that depression patients often show reduced bone density, which may be related to a disruption in the nervous system’s regulation of bone metabolism[114]. Neurotransmitters (such as serotonin, norepinephrine) and neurohormones (such as cortisol) play key roles in the onset of depression[115]. These substances not only affect emotional regulation in the brain but also influence bone remodeling and metabolism through neural–brain interactions. Research has shown that the sympathetic nervous system in depression patients is often overactive, which suppresses osteoblast function through the bone–brain axis, promoting bone resorption and leading to osteoporosis[116]. Additionally, chronic inflammation in depression patients may further disrupt bone metabolism through neuro-immune interactions, exacerbating bone health issues[117].

#### Relationship between Alzheimer’s disease and the bone–brain axis

Alzheimer’s disease (AD), a prevalent neurodegenerative disorder, demonstrates escalating incidence rates paralleling global demographic aging trends[118]. Contemporary research identifies substantial associations between AD pathophysiology and bone–brain axis dysregulation[119]. Osteocalcin and bone morphogenetic proteins (BMPs), as skeletal-derived signaling mediators, exert regulatory influences extending beyond osseous metabolism to encompass cerebral cognitive modulation[41]. Experimental evidence suggests osteocalcin enhances neuronal viability and synaptic adaptability, whereas diminished bone mineral density may correlate with AD progression[2].Age-related degenerative processes associated with global population aging potentially exacerbate cognitive deterioration through impaired skeletal-neural crosstalk mechanisms[50]. The frequent comorbidity of osteoporosis and AD likely stems from shared pathophysiological pathways involving systemic inflammation and endocrine fluctuations[120].

#### Relationship between the bone–brain axis and cancer

Certain cancer patients, particularly those with bone metastasis, exhibit symptoms of bone metabolism disorders[121]. In these patients, the bones are not only invaded by tumor cells but are also regulated by the nervous system. Tumor growth and metastasis may activate the sympathetic nervous system, promoting bone resorption and the development of osteoporosis[122]. Moreover, the systemic inflammatory response induced by cancer may also affect bone metabolism through neuro-immune pathways. At present, research has shown that the imbalance of this axis is related to the progression of cancer, especially in bone metastasis and neuroinflammation^[123,124]^. A key player is the RANKL signaling pathway, which regulates bone resorption by activating osteoclasts. In cancer, tumor cells hijack RANKL signaling to promote bone destruction. Tumor-secreted factors like PTHrP and IL-6 upregulate RANKL in osteoblasts, creating a “vicious cycle” of bone resorption and tumor growth. Beyond bone, RANKL may influence the brain by exacerbating neuroinflammation, potentially contributing to cognitive impairment in cancer patients. The brain also regulates RANKL expression through neural pathways. For example, stress-induced sympathetic activation increases RANKL production, further driving bone resorption. This bidirectional interplay highlights the complex role of the bone–brain axis in cancer. Advances in traditional medicine in cancer treatment have demonstrated its potential in cancer treatment, such as by modulating neuroimmune pathways or targeting RANKL signaling to alleviate symptoms associated with bone metastasis[125].

#### Role of the bone–brain axis in fracture healing

Fracture repair constitutes a sophisticated physiological phenomenon requiring multisystem coordination among osseous structures, cartilaginous components, and neural networks[126]. Contemporary research confirms the nervous system’s fundamental role as a biological regulator during osseous regeneration[127]. Successful bone reconstitution necessitates not only tissue reconstruction but also neurotrophic modulation, particularly through the regulatory contributions of nerve growth factor (NGF) and brain-derived neurotrophic factor (BDNF) in healing cascades[128]. The nervous system promotes fracture healing by regulating neurotransmitter release and neuroinflammation, among other mechanisms[127].

#### Relationship between the bone–brain axis and other diseases

Pathological alterations in the bone–brain axis demonstrate pathophysiological associations with multiple systemic disorders including metabolic syndrome components and cardiovascular pathologies[2]. These pathologies exert dual impacts on osseous metabolism while mediating skeletal deterioration via neural regulatory pathways. Clinical populations with metabolic disorders frequently exhibit impaired bone remodeling and osteoporotic manifestations, whereas cardiovascular disease progression correlates with compromised skeletal integrity due to sustained hemodynamic and glycemic dysregulation[129].

### Exercise and the bone–brain axis

Exercise is not only significant for bone health but also plays a crucial role in promoting neurological function, delaying aging, and preventing diseases through the mechanisms of the bone–brain axis[130]. Recent studies have shown that the interaction between exercise and the bone–brain axis can affect bone metabolism, neuronal plasticity, and cognitive function, providing essential evidence for the link between bone health and brain health^[27,50]^.

#### The impact of exercise on bones

Physical activity constitutes a critical determinant in optimizing osseous density and structural integrity[131]. Through weight-bearing exercises (such as running, jumping, weightlifting, etc.), bones can bear significant mechanical loads, stimulating osteoblast activity and promoting the synthesis and mineralization of the bone matrix, thereby increasing bone density and strength[132]. Regular exercise enhances the bones’ ability to withstand pressure and improves bone resilience, reducing the risk of osteoporosis and fractures[133]. The metabolic influence of exercise on skeletal tissue extends beyond biomechanical loading effects to encompass sophisticated neuroregulatory mechanisms[134]. Exercise-induced skeletal mediators including osteocalcin and osteogenic differentiation factors facilitate bone–brain communication during physical exertion[130]. Scientific evidence demonstrates that exercise-mediated bone density improvements exert indirect neuromodulatory effects, particularly through upregulating neurotrophic factors (e.g., BDNF) and neuroprotective agents to stimulate neural restoration processes[135].

#### The impact of exercise on brain function

Extensive research substantiates exercise-induced neurocognitive benefits. Physical activity augments cerebrovascular perfusion and oxygenation, facilitating neuronal preservation and synaptic adaptability[136]. Concurrently, exercise potentiates neurotrophic signaling pathways, particularly BDNF synthesis, which is fundamental for neuronal circuit development and maintenance[137]. Systematic modulation of exercise parameters (intensity, frequency, duration) demonstrates significant cognitive enhancement, deceleration of age-related degeneration, and mitigation of AD progression[138]. Beyond cognitive optimization, exercise improves affective regulation, effectively reducing anxiety/depression susceptibility[139].

#### The role of the bone–brain axis in exercise

**Figure 3.**
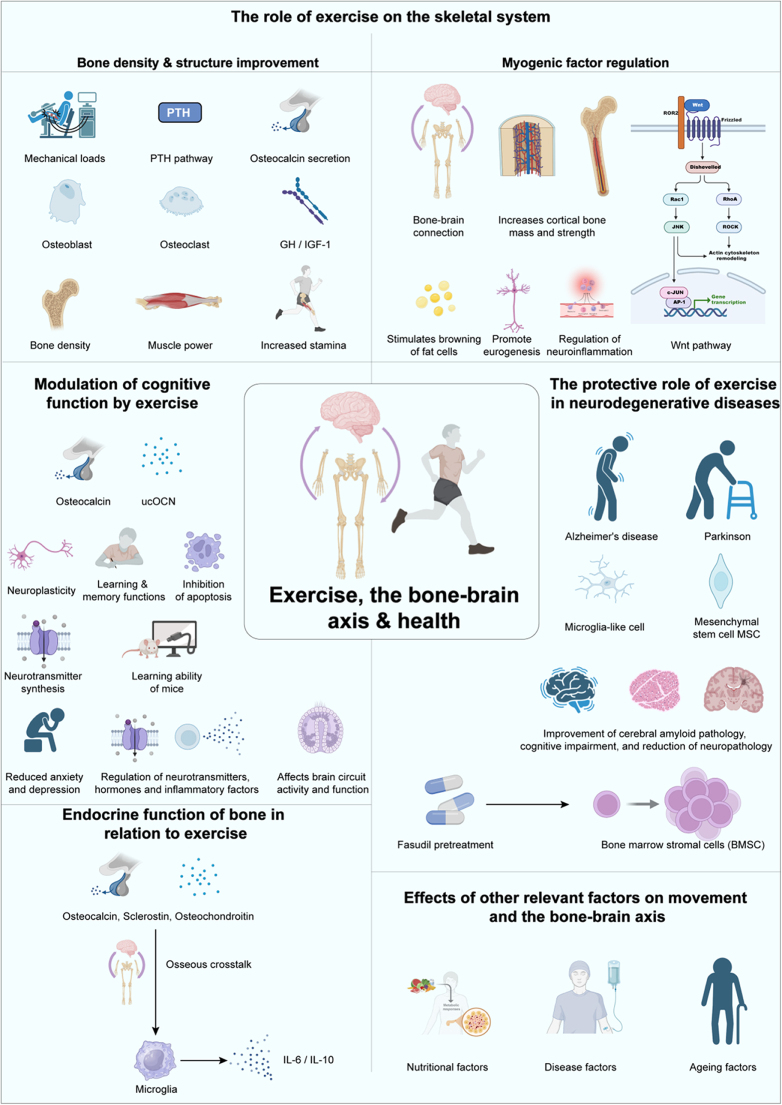
The multifaceted role of exercise in promoting skeletal, cognitive, and neuroprotective health through the bone–brain axis.

The impact of exercise on the bone–brain axis is bidirectional[41]. On the bone side, exercise stimulates the osteogenic process through mechanical loading and communicates with the brain via bone-derived factors[3]. On the brain side, exercise can promote the regulation of bone metabolism by activating neural pathways. For example, exercise increases the secretion of neurotransmitters and enhances neuroplasticity, modulating the bone marrow microenvironment and thus promoting bone health[140]. A study has shown that exercise can regulate bone remodeling through the nervous system, playing an essential role in the bone–brain axis mechanism. Exercise enhances the coordination between the nervous system and bone metabolism, promoting the secretion of factors like osteocalcin in bones, which in turn positively affects cognitive function in the brain (Fig. 3,Table 1)^[3,50]^. Furthermore, exercise improves neuroinflammation and reduces oxidative stress, thereby slowing neurodegenerative diseases associated with bone degeneration[141].

This figure comprehensively demonstrates the systemic effects of exercise on the skeletal system and its influence on brain health via the bone–brain axis. Exercise enhances bone density and structure through mechanical loading, activation of the PTH pathway, and osteocalcin secretion. It stimulates osteoblast activity, suppresses osteoclast function, and increases muscle power and stamina through the GH/IGF-1 pathway.Exercise-induced regulation of myogenic factors promotes cortical bone mass and strength, browning of fat cells, neurogenesis, and the suppression of neuroinflammation via pathways such as Wnt signaling. The figure also highlights the endocrine role of bone in response to exercise, where osteocalcin, sclerostin, and osteochondrodin mediate crosstalk between bone and brain, influencing neurotransmitter synthesis, inflammatory cytokines (IL-6 and IL-10), and microglial activity.Cognitive modulation by exercise is depicted through its effects on neuroplasticity, learning, and memory, and its inhibition of apoptosis in neuronal cells. Exercise reduces anxiety and depression by regulating neurotransmitters, hormones, and inflammatory pathways, thus improving brain circuit activity and function. Additionally, exercise provides neuroprotection against neurodegenerative diseases such as Alzheimer’s disease and Parkinson’s disease by improving amyloid pathology, cognitive impairment, and neuropathy through mechanisms involving mesenchymal stem cells (MSCs) and microglia-like cells.Lastly, the figure considers external factors, such as nutrition, disease, and aging, that influence the bone–brain axis and overall physiological health, reinforcing the pivotal role of exercise in mitigating these impacts.

#### The role of exercise in neurodegenerative diseases

In neurodegenerative pathologies including AD and PD, impaired bone–brain axis communication frequently correlates with diminished skeletal mineral density and cognitive deterioration^[142]^. As a non-pharmacologic intervention, structured physical regimens demonstrate significant therapeutic efficacy in enhancing life quality for affected patients[143].Exercise-induced elevation of osteocalcin and related osseous mediators confers dual benefits for skeletal integrity and neurocognitive enhancement, particularly memory consolidation[26]. For Alzheimer’s cohorts, routine exercise modulates disease progression through tripartite mechanisms: optimizing cerebral hemodynamics, amplifying neurotrophic signaling, and suppressing neuroinflammatory cascades[144]. These exercise-mediated regulatory effects on skeletal-neural crosstalk establish novel investigative pathways for preclinical intervention in neurodegeneration.

#### The impact of exercise on the bone–brain axis in older adults

With aging, both bone and brain health gradually decline, and the function of the bone–brain axis is also affected. Research demonstrates that structured physical regimens in geriatric populations substantially enhance skeletal integrity, decelerate osteoporotic progression, and optimize cognitive performance through bone–brain axis modulation[145]. By increasing physical activity, older adults can not only enhance bone density and reduce fracture risk but also improve memory and cognitive ability, slowing the rate of cognitive decline[146]. Furthermore, exercise can alleviate chronic inflammation and reduce oxidative stress, thereby further slowing the degeneration of the bone–brain axis during aging[147].

#### The relationship between exercise types and the bone–brain axis

Exercise modality differentially impacts skeletal-neural interactions: weight-loading activities (e.g., ambulation, sprinting, vertical jumps) effectively promote osteogenic stimulation and mineral density augmentation, whereas cardiovascular training (e.g., aquatic exercises, stationary cycling, choreographed movement) primarily enhances cardiocerebrovascular efficiency and neural functionality[148]. Scientific evidence confirms combined aerobic-resistance training protocols exert maximal bidirectional regulatory effects on skeletal-neural communication networks[149].

## Effects of exercise on the skeletal system

### Exercise and improvement of bone density and bone structure

Exercise improves bone density and bone structure not only through direct mechanical load on the skeletal system but also through indirect mechanisms such as endocrine regulation (Table 2)[150]. Through these mechanisms, exercise effectively promotes bone health, enhances bone density, improves bone structure, and increases bone strength and resilience, thereby reducing the risk of osteoporosis[151].

**Table 2:** Effects of exercise on the skeletal system

Mode of action	Target	Mechanism	Effect	Example
Direct mechanical charge action	Osteoblasts	Complex signaling pathways	Increases bone matrix synthesis and enhances bone mineralization	-
Promote the secretion of bone-derived factors	Osteoblasts, mature bone cells	endocrine	Promote bone metabolism and enhance bone strength	Osteocalcin, Sclerostin
Neuromuscular electrical stimulation (NMES)	Muscle	Increase muscle strength	Improve bone density and restore mobility	-
Promote the secretion of myogenic factors	Muscle	Endocrine	Improve bone metabolism environment and enhance bone metabolism activity	Irisin, myosin
Hormone regulation	Parathyroid gland	Endocrine	Improve bone metabolism and enhance bone health	PTH
	Pituitary	Endocrine	Increase muscle mass and improve the weight-bearing capacity of bones	GH
Regulates calcium balance	-	Enhances the activity of vitamin D	Promote calcium absorption and further improve bone density	-
Sympathetic nervous system regulation	Sympathetic nerves	Regulates plasma catecholamine levels and sympathetic adrenal activity	Promote bone metabolism and alleviate degenerative changes in aging bones	-

#### Direct effect of mechanical load

Mechanical load induced by exercise is one of the core stimuli for bone health[152]. By applying pressure to the bones through weight-bearing exercises, strength training, and other forms, the self-repair and remodeling of bones are triggered[153]. Osteoblasts are sensitive to mechanical load and can regulate the balance between bone formation and resorption through a series of complex signaling pathways[154]. When osteoblasts are stimulated by mechanical load, they initiate the bone repair mechanism, increase the synthesis of bone matrix, and enhance the mineralization of bone tissue, thereby increasing bone density and strength[155]. This mechanical load stimulation not only enhances bone density but also improves the structure of trabecular bone, making it more robust and stable[156]. Studies have found that weight-bearing exercises significantly improve bone density in the lumbar spine, femur, and other major skeletal sites, especially in postmenopausal women, where the improvement in bone density is particularly notable[157]. For instance, strength training, running, and other impact exercises effectively increase bone density in postmenopausal women, reducing the risk of fractures. Postmenopausal women who underwent 12 months of resistance training had a 6.3% increase in femoral neck BMD and a 28% decrease in fracture risk[158].

#### Secretion and function of bone-derived factors

The skeletal system functions as both a biomechanical framework and a critical endocrine regulator, synthesizing multiple bioactive mediators[159]. Physical activity stimulates osseous metabolic activity, enhancing secretion of key regulators including osteocalcin (OCN) and sclerostin (SOST)[160]. Empirical data confirm exercise-induced OCN elevation significantly enhances osseous metabolic turnover and structural resilience[161]. As a Wnt/β-catenin pathway antagonist, SOST modulates osteoblastic activity and remodeling processes. Emerging studies indicate SOST may mediate neuroimmune interactions via bone marrow macrophage communication, potentially affecting neuroinflammatory pathways and affective state modulation[162]. Exercise-mediated SOST regulation promotes osteogenesis while suppressing resorption, thereby optimizing skeletal integrity[133]. Concurrently, exercise activates Wnt-β-catenin signaling to facilitate adaptive bone remodeling and mechanical load responsiveness[163]. This dual regulatory mechanism not only improves osseous architecture but also exerts systemic homeostatic effects, particularly through neuromodulatory pathways.

#### Specific impact of exercise types on bone health

Exercise modalities differentially influence osseous density and microarchitecture optimization. Weight-loading activities, resistance protocols, and impact training demonstrate substantial bone mineral density (BMD) enhancement[164]. For postmenopausal populations, non-weight-bearing resistance interventions particularly improve femoral and tibial BMD, whereas combined exercise strategies show superior efficacy in spinal density augmentation[165]. Cardiovascular training and dance-based regimens effectively mitigate bone mass depletion, crucially reducing fracture susceptibility[166]. Longitudinal studies document significant lumbar BMD improvement with concurrent trabecular thickness and mineral density enhancement in weight-bearing bones following structured resistance programs[167]. Mechanistically, physical activity stimulates bone marrow mesenchymal stem cell proliferation, driving osteogenic differentiation to accelerate osseous regeneration[167].

#### The auxiliary role of Neuromuscular Electrical Stimulation (NMES)

Beyond conventional exercise modalities, neuromuscular electrical stimulation (NMES) has demonstrated therapeutic efficacy for patients with restricted mobility due to pathological conditions or physical limitations[168]. NMES enhances muscle strength and endurance through electrical stimulation, significantly reducing muscle atrophy during prolonged periods of inactivity or immobilization[169]. Research has shown that NMES is superior to traditional voluntary training in reducing muscle strength loss during immobilization, particularly when patients are in a cast[151]. In fixed patients, NMES can reduce muscle atrophy by 15% and improve trabecular volume by 12%. In the skeletal system, NMES enhances the strength of thigh and gluteal muscles, further improving the patient’s mobility. Especially for patients with bone metastasis or other serious conditions, NMES can serve as an effective palliative treatment, improving their quality of life[170]. By increasing muscle strength, NMES not only improves bone density but also helps patients regain mobility, alleviating the discomfort caused by bone system involvement[171].

### Myokines in the bone–brain axis

The skeletal-neural interplay continues to gain scientific prominence, particularly regarding exercise-mediated dual regulatory impacts on osseous and neurological systems. Exercise-induced molecular mediators, particularly myokines like irisin and myostatin, demonstrate multilevel regulatory capacity within bone–brain connectivity networks (Table 2)[69]. Through these bioactive agents, physical activity not only enhances skeletal homeostasis but also exerts neuromodulatory effects, establishing a bidirectional regulatory circuit between osseous and neural tissues.

#### Role of Irisin

Irisin, an exercise-induced myokine released from skeletal muscle tissue, serves critical functions in energy homeostasis regulation and osseous mineralization enhancement[69]. Research confirms its capacity to optimize the skeletal metabolic milieu through facilitation of white-to-brown adipose tissue conversion, thereby augmenting bone remodeling activity[75]. Additionally, Irisin regulates the activity of bone cells, promoting the increase in bone density and the remodeling of bone tissue[75]. Irisin not only directly affects the skeletal system but also influences the nervous system, regulating brain function, particularly in protecting brain health after exercise[53]. In the bone–brain axis, Irisin may promote the secretion of neurotrophic factors such as BDNF through its regulation of bone metabolism, further enhancing the survival and growth of neurons[172]. The secretion of Irisin increases with exercise intensity, indicating that it bridges the relationship between bone and brain health[173].

#### Myosin and bone–brain interaction

Myosin, the principal contractile protein in striated muscle fibers, mediates mechanical transduction during physical exertion. Through its regulation of muscular contraction dynamics and metabolic adaptation, myosin indirectly modulates skeletal system responses to mechanical loading. The activity of myosin stimulates the differentiation of bone marrow mesenchymal stem cells into osteoblasts, promoting an increase in bone density and structural remodeling[174]. Additionally, myosin enhances muscle strength, improving mechanical load support on the skeleton and further promoting bone health[175]. The secretion of myosin is closely related to muscle activity, and its effects on the skeleton increase with exercise intensity. Myosin enhances osseous load adaptability, thereby facilitating bone–brain system interactions that synchronously improve skeletal integrity and cognitive performance[3]. Scientific investigations confirm exercise-induced myosin upregulation not only augments osseous mechanical resilience but also optimizes neural functions, substantiating exercise’s multidimensional benefits on bone–brain axis homeostasis[50].

#### Bidirectional regulation of bone–brain health

Physical activity establishes a reciprocal regulatory framework between osseous and neural health through myokine-mediated signaling within the bone–brain axis^[89,176]^. The skeleton, through the secretion of factors such as osteocalcin and Irisin, promotes not only the health of the bones themselves but also improves brain function by regulating the nervous system, thus enhancing cognitive abilities[50]. Conversely, a healthy skeletal system, through the regulation of factors like PTH, improves bone density, strengthens bones, and provides better support for the brain, creating a healthy cycle of bone–brain interaction[1]. This bidirectional regulatory mechanism not only explains how exercise promotes dual health in both bones and the brain but also provides a theoretical foundation for the application of exercise interventions in diseases such as osteoporosis and dementia. The secretion of myokines induced by exercise helps maintain the stability of both the skeleton and the nervous system, slowing down bone loss and cognitive decline during aging, with important clinical significance.

### Regulation of bone endocrine functions by exercise

The skeletal system functions beyond biomechanical support as a dynamic endocrine regulator, synthesizing and releasing multiple bioactive mediators including osteocalcin (OCN) and sclerostin (Table 2)[33]. These skeletal-derived factors critically maintain systemic metabolic equilibrium, osseous remodeling balance, and holistic physiological homeostasis. Exercise modulates the secretory profile of these endocrine mediators, thereby enhancing osseous integrity through mineral density augmentation and microarchitectural optimization[177].

#### Regulation of osteocalcin and sclerostin

Osteocalcin and sclerostin are two important bone-derived hormones secreted by mature osteocytes[33]. Osteocalcin is closely related to physiological processes such as glucose metabolism and energy balance[178]. Research demonstrates physical activity markedly enhances osteocalcin production, optimizing skeletal mineralization and structural integrity while stimulating adipose tissue lipolysis for body fat regulation[179]. Sclerostin, a Wnt/β-catenin pathway inhibitor predominantly secreted by osteocytes, critically regulates osseous remodeling processes[162]. Exercise may help reduce sclerostin levels (10%-50%), which could improve bone density and strength[180]. Therefore, through regulating these bone-derived factors, exercise not only enhances skeletal health but may also have a profound impact on overall metabolism, energy balance, and the endocrine system.

#### Parathyroid hormone (PTH) and bone adaptation

Parathyroid hormone (PTH) is one of the most important hormones in bone metabolism, playing a critical role in maintaining calcium homeostasis by promoting bone resorption and remodeling[181]. Exercise influences PTH secretion, thereby promoting adaptive bone responses[182]. Intermittent weight-bearing exercise significantly elevates PTH synthesis, which activates osteoblastic proliferation and bone formation[183].The PTH signaling cascade involves RANKL-mediated osteoclastic activation through nuclear factor-κB pathway engagement, coordinating bone remodeling dynamics[184]. Exercise-induced PTH1 receptor upregulation amplifies osteoblastic responsiveness to hormonal signals, accelerating matrix deposition and structural adaptation[185]. Mechanistic studies confirm exercise-driven PTH elevation increases osteocalcin and collagen gene expression, essential for enhancing bone density and biomechanical competence[182]. Therefore, exercise improves bone metabolism through modulation of the PTH signaling pathway, enhancing skeletal health, particularly in preventing osteoporosis and reducing fracture risk[177].

#### Growth Hormone (GH) and bone metabolic regulation

Growth hormone (GH) serves critical regulatory functions in skeletal development and maturation[186]. GH induces hepatic insulin-like growth factor 1 (IGF-1) synthesis, exerting pleiotropic effects on osseous, muscular, and systemic tissue homeostasis[187]. Exercise, especially high-intensity strength training and weight-bearing exercises, effectively stimulates GH secretion, which through the GH-IGF-1 signaling pathway, enhances bone formation and increases bone density[188]. Studies have shown that GH plays a crucial role in bone regulation. By activating the IGF-1 signaling pathway, GH promotes bone matrix synthesis and enhances osteoblast function[189]. GH replacement therapy has also been proven to effectively alleviate bone loss caused by GH deficiency, especially in elderly individuals and those with growth hormone deficiency during childhood, with significant improvements in bone density[190]. Additionally, GH interacts with exercise by regulating fat metabolism and muscle growth. GH promotes muscle synthesis, increasing muscle mass, which enhances the skeleton’s ability to support weight, further promoting bone health[191].

#### Hormonal regulation and exercise’s impact on calcium balance

The effects of exercise on skeletal health are not limited to the regulation of bone-metabolic hormones but also involve maintaining calcium balance[192]. Calcium is a fundamental element for bone health, and its intake, metabolism, and utilization directly influence bone density and strength[193]. Exercise, especially weight-bearing and strength training, increases calcium utilization in the body, promoting calcium deposition and enhancing bone density[194]. The impact of exercise on calcium balance occurs through several mechanisms. First, exercise enhances PTH secretion, promoting calcium release and reabsorption to maintain blood calcium homeostasis[182]. Second, exercise increases the activity of vitamin D, facilitating calcium absorption, further improving bone density[195]. Vitamin D insufficiency and suboptimal calcium intake constitute primary risk factors for osteoporosis pathogenesis and fracture susceptibility[196]. Therefore, combining exercise with proper nutrition can effectively regulate calcium balance and improve bone health.

#### Exercise’s overall regulation of the neuro-endocrine–brain system

Exercise not only regulates the skeletal system but also interacts closely with bone metabolism through the neuro-endocrine system[134]. Exercise modulates the secretion of neurotransmitters, hormones, and inflammatory factors, enhancing the health of the nervous system, improving cognitive function, and promoting improvements in bone density and structure[11]. For instance, the effects of exercise on skeletal health are mediated not only through the regulation of hormones such as osteocalcin and sclerostin but also by increasing the secretion of neurotransmitters like Irisin, which promotes neuroprotection and neuroregeneration, enhancing brain function[69]. Exercise’s endocrine regulation of the bones is manifested through complex interactions between the bone, nervous, and endocrine systems[197]. These physiological interactions positively modulate bone remodeling dynamics and mineral density while exerting systemic neurometabolic regulatory effects, underscoring exercise’s multidimensional health benefits.

### The effects of exercise on bone metastasis and osteoporosis

Bone metastatic complications in malignancies (breast/prostate/lung carcinomas) frequently manifest as pathological fractures, chronic pain, and functional impairment, severely compromising patient outcomes[198]. Osteoporotic conditions, marked by diminished bone mass and structural fragility, disproportionately affect postmenopausal and geriatric populations[199]. Structured exercise protocols demonstrate therapeutic efficacy in both metastatic bone disease mitigation and osteoporosis management by enhancing biomechanical competence, reducing fracture incidence, and optimizing skeletal mineralization[200]. Additionally, it improves protein metabolism and nutritional status in cancer patients, addressing issues like hypoalbuminemia, which worsens prognosis in advanced cancer[201]. Exercise also helps maintain bone health by regulating calcium balance and preventing hypercalcemia, a common tumor-related disturbance.

In osteoporosis management, exercise stimulates osteoblast activity and promotes bone formation through mechanical loading[202]. Weight-bearing and strength training, especially in postmenopausal women, significantly increase bone density and reduce bone loss[203]. Aerobic exercise also helps preserve bone density and reduce fracture risk in the elderly[204]. Furthermore, exercise activates mesenchymal stem cells, inhibits osteoclast activity, and may reduce osteoporosis incidence[205]. In bone metastasis, exercise improves circulation and immune function, potentially slowing tumor spread within bones[206]. Studies in breast cancer have shown that regular exercise reduces bone-related events, alleviates pain, and enhances both bone metabolism and functional capacity[207]. Exercise, a safe and effective intervention, supports the comprehensive management of patients with bone metastasis and osteoporosis[200].

### Interaction between aging bone and the sympathetic nervous system

Aging leads to bone density loss due to reduced osteocyte activity, fewer osteoprogenitor cells, and decreased calcium responsiveness, increasing osteoporosis and fracture risks[208]. The aging process significantly impacts sympathetic nervous system functionality, which serves a pivotal regulatory function in osseous metabolic processes[92]. Scientific investigations confirm sympathetic activity modulates bone remodeling through catecholamine level regulation and sympathetic-adrenal axis interactions[209].

In aging bones, sympathetic nerve density increases, particularly in the periosteum, although the number of tyrosine hydroxylase (TH)-positive fibers decreases, suggesting altered sympathetic function[210]. This nerve density increase correlates with thinning of the periosteum, which can impact osteocyte function[211]. The sympathetic nervous system affects osteoblast activity through adrenergic receptors, particularly β2-adrenergic receptors (β2AR)[212]. Studies show that inhibiting β2AR enhances osteogenic responses under mechanical load, suggesting that blocking β2AR may promote periosteal adaptation and improve osteoblast function[213]. Moreover, β2AR-deficient mice exhibit stronger bone responses to mechanical stimuli, further supporting β2AR’s role in bone metabolism[214]. Moderate exercise enhances bone health in aging populations through multiple mechanisms, including improving bone adaptation to mechanical load and regulating sympathetic nervous activity (Table 2)[215]. Combining exercise with pharmacological interventions, such as β2AR antagonists, could further promote bone metabolism and increase bone density[216]. Exercise-induced mechanical stimulation, combined with sympathetic regulation, improves bone mass and structure, helping prevent osteoporosis[202]. In conclusion, the interplay between aging bones and the sympathetic nervous system highlights the complexity of bone adaptation. Combining exercise with pharmacological treatment could restore periosteal adaptation, regulate sympathetic activity, and alleviate bone degeneration in the elderly, providing effective strategies for bone health management in aging populations.

## Effects of exercise on brain health

### The relationship between physical activity and the bone–brain axis

The bone–brain axis constitutes a bidirectional communication network where skeletal-derived mediators (OCN, SOST, osteoprotegerin/OPG) traverse the BBB to regulate neurodevelopmental processes, cognitive operations, and affective states^[41,217]^. Among these, osteocalcin is a key link between the skeleton and the brain. Secreted by osteocytes, osteocalcin enhances synaptic plasticity, memory, and learning by binding to neuronal receptors such as Gprc6a, especially in regions like the hippocampus[218]. Osteocalcin enhances cognitive performance via activation of Wnt/β-catenin signaling pathways essential for synaptic plasticity and mnemonic consolidation (Fig. 3)[219]. Clinically, diminished osteocalcin concentrations correlate with cognitive deterioration and AD progression, where plasma OCN levels demonstrate significant depletion[220].

**Figure 4.**
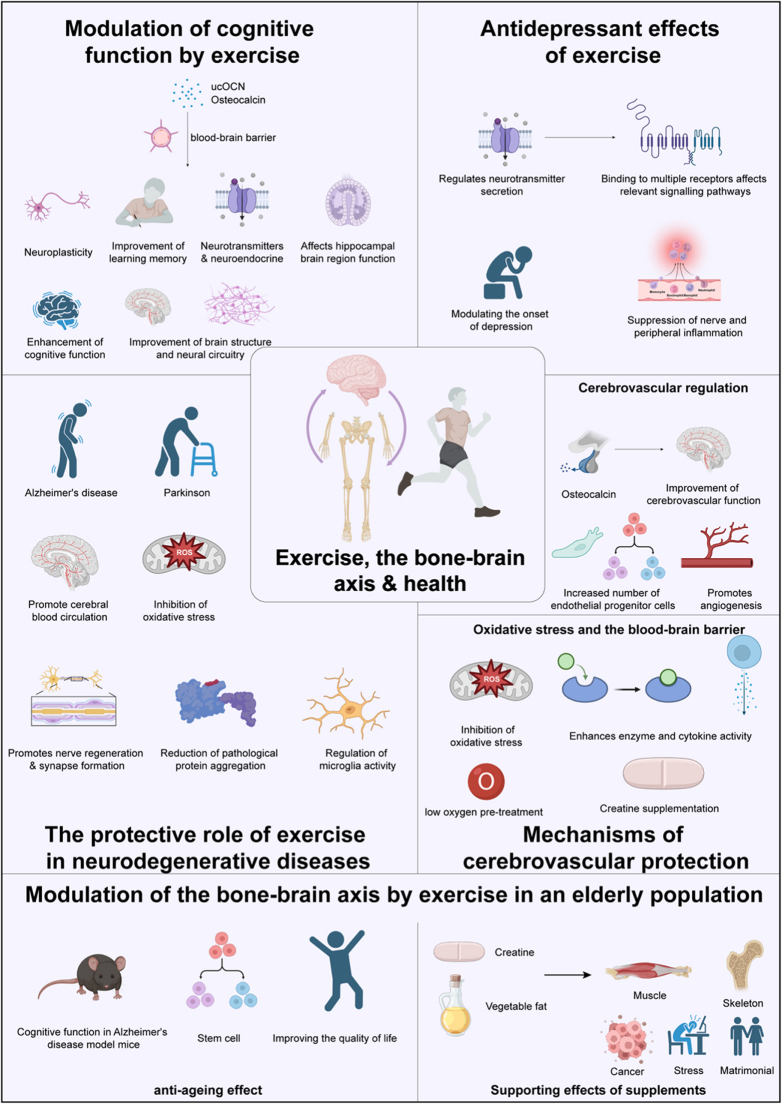
The comprehensive impact of exercise on the bone–brain axis, cognitive function, cerebrovascular health, and neuroprotection.

Physical exercise enhances osseous integrity while modulating bone–brain axis functionality, exerting neuromodulatory effects through direct and indirect pathways^[76,130]^. Studies show that regular exercise increases osteocalcin levels, which, via the bloodstream, enter the brain and improve cognitive function and emotional well-being[50]. For example, long-term aerobic exercise has been shown to boost osteocalcin levels and improve cognitive abilities, particularly in older adults^[221,222]^. This interaction between exercise and the bone–brain axis may contribute to mitigating cognitive decline and neuropsychiatric disorders like depression (Fig. 4)[42]. Physical activity stimulates the release of skeletal-derived mediators including fibronectin type III domain-containing protein 5 (FNDC5), which potentiates BDNF activity to augment neuronal regeneration and synaptic adaptability, thereby reinforcing cognitive performance. Exercise-induced peroxisome proliferator-activated receptor gamma coactivator 1-alpha (PGC-1α) upregulation initiates FNDC5 biosynthesis, with subsequent proteolytic processing in striated muscle tissue generating the bioactive myokine irisin[223]. This molecular cascade presents novel therapeutic avenues for neurodegenerative pathology intervention. Furthermore, bone marrow-derived mesenchymal stem cells (MSCs) participate in skeletal-neural communication networks[224]. These pluripotent cells undergo transdifferentiation into microglial analogs capable of secreting neuroprotective mediators that suppress neuroinflammatory responses and enhance neuronal viability[225]. MSCs exhibit a microglial like phenotype under neuroinflammatory conditions, characterized by the expression of typical microglial markers. For example, Iba1 is upregulated during MSC differentiation, leading to remodeling of the cytoskeleton to obtain phagocytic activity. The fractalkine receptor CX3CR1 is crucial for the migration of MSCs to the site of brain injury and is induced during differentiation. In addition, MSCs migrate to the brain through chemotactic signals and adhesion molecules. Research has found that exercise enhances the mobilization and differentiation of MSCs. Aerobic training can increase SDF-1 levels and promote MSC migration to the brain. Resistance exercise upregulates CX3CR1 in MSCs, enhancing their neuroprotective ability. For instance, bone marrow-derived microglia-like cells have been shown to alleviate amyloid pathology and cognitive impairments in AD mouse models, suggesting that bone marrow stem cells contribute to both bone health and immune regulation in the nervous system[226].

This figure illustrates the multifaceted effects of exercise on brain health through the bone–brain axis. Exercise-induced factors, such as ucOCN and osteocalcin, cross the BBB to modulate cognitive functions, including neuroplasticity, neurotransmitter synthesis, and hippocampal region activity. These effects enhance learning and memory, improve brain structure and neural circuitry, and alleviate symptoms of Alzheimer’s and Parkinson’s diseases by promoting cerebral blood circulation, inhibiting oxidative stress, reducing pathological protein aggregation, and regulating microglial activity .The antidepressant effects of exercise are depicted through the regulation of neurotransmitter secretion, modulation of receptor signaling pathways, and suppression of neuroinflammation. Exercise also improves cerebrovascular health by increasing osteocalcin levels, promoting angiogenesis, enhancing endothelial progenitor cell numbers, and improving vascular function. Furthermore, the figure highlights the role of exercise in reducing oxidative stress, which enhances enzymatic and cytokine activity, and stabilizes the BBB. Supplementary strategies such as creatine supplementation and dietary fat intake further support these benefits. Exercise-mediated bone–brain axis modulation in elderly populations improves life quality by promoting cognitive functions, stem cell activity, and anti-aging effects. This visual representation underscores exercise as a central intervention for maintaining skeletal, cognitive, and cerebrovascular health while providing protection against neurodegenerative diseases.

Exercise enhances bone health and the bone–brain axis, indirectly benefiting brain health, particularly cognitive function and emotional regulation (Table 3)[50]. In neurodegenerative diseases like AD and PD, exercise improves the brain’s microenvironment, promotes neural plasticity, and has potential therapeutic effects by increasing bone-derived factors[227]. For example, research has shown that bone marrow stromal cell transplantation can improve cognitive dysfunction in AD mouse models, and an increase in osteocalcin levels may protect through the bone–brain axis[227]. In summary, exercise improves bone density and metabolism while boosting the nervous system, potentially slowing the progression of neurodegenerative diseases and enhancing brain health.

**Table 3:** The role of exercise in brain health

	Effector	Example	Mechanism of action	Effect
Regulate cognitive function	Bone derived factor	Osteocalcin	Modulating neuroplasticity to enhance synaptic plasticity	Maintain normal cognitive function
		Uncarboxylated osteocalcin	Crossing the blood–brain barrier affects the secretion of nerve growth factor	Promotes the health and function of brain neurons
	Neurotrophic factors	BDNF	Promotes nerve growth, dendrite growth and synaptic strengthening	Improve cognitive function
	Anti-inflammatory factors	IL-10	Reduce inflammation	Delaying cognitive decline
Slowing down neurodegenerative diseases	Bone derived factor	Osteocalcin	Regulates hippocampal-dependent memory and promotes synaptic plasticity and memory enhancement via the Wnt-β-catenin signaling pathway	Promoting cognitive recovery in patients with neurodegenerative diseases
	Bone marrow mesenchymal stem cells	-	Enhance neuroplasticity and improve synaptic function	Improved brain amyloid plaque pathology in a mouse model of Alzheimer’s disease
	Bone marrow stromal cells	-	Protects the dopaminergic nervous system	Alleviating neurodegeneration in a mouse model of Parkinson’s disease
	Bone marrow-derived microglia-like cells	-	Slow down the progression of neuroinflammation and neurodegeneration	Improve the pathological state of Parkinson’s disease animal models
Mood regulation and antidepressant effects	Bone derived factor	Osteocalcin	Regulate emotions and cognitive abilities	Low levels of osteocalcin are significantly negatively correlated with negative emotional states such as anxiety and depression
		Uncarboxylated osteocalcin	Regulates the synthesis of neurotransmitters, improves neuroplasticity, and reduces inflammatory responses	Significant antidepressant effect
	Endocrine hormones	Cortisol	Reduces over-activation of the HPA axis	Relieve symptoms of depression caused by stress
	Anti-inflammatory factors	IL-10	Reduce inflammation	Improve depression symptoms
	Microglia	-	Reducing the inflammatory burden in the brain	Positive impact on the treatment of depression
Improve cerebrovascular health	Biomarkers	Irisin	Regulate metabolic health, improve cardiovascular function, regulate neuroplasticity	Reduce the risk of cerebrovascular disease
		Growth hormone	Promotes the secretion of insulin-like growth factor (IGF-1) by the liver	Maintain neuronal function, improve cerebral blood flow and enhance angiogenesis
	Microvascular network	-	Promote endothelial cell proliferation and secretion of angiogenic factors	Promote the expansion of brain microvessels and increase cerebral blood flow

### The modulatory effects of physical activity on cognitive function

Age-related cognitive deterioration progresses naturally, yet structured aerobic exercise demonstrates recognized efficacy in attenuating cognitive degradation and optimizing neural performance[228]. Physical engagement enhances cognitive capacities across demographics, with pronounced effects in younger populations and cognitively compromised individuals through neuroplastic adaptation, neurotrophic upregulation, and bone–brain axis-mediated neural optimization[229].

The impact of aerobic exercise on cognition varies across age groups. In adolescents and middle-aged adults, exercise notably boosts attention, memory, and learning abilities. It strengthens neural connections and synaptic plasticity, crucial for memory and learning[140]. However, diminished therapeutic returns are observed in the 60–80 age cohort, potentially attributable to suboptimal exercise modality selection and intensity calibration[230]. Older adults may need more intense or specific interventions for noticeable cognitive improvements[231]. Despite this, aerobic exercise remains an accessible, low-cost intervention for improving cognitive function in older adults[232]. Bone-derived factors, such as OCN and ucOCN, play an essential role in regulating cognitive function[2]. These factors, involved in bone remodeling, also promote synaptic plasticity and cognitive functions like memory and learning. Studies show that osteocalcin enhances synaptic plasticity by modulating neuroplasticity, which is critical for memory formation and learning efficiency[26]. Mice lacking osteocalcin exhibit cognitive impairments, indicating its vital role in normal cognitive function[219]. Osteocalcin (OCN) and undercarboxylated osteocalcin (ucOCN) traverse the blood-brain interface, modulating neurotrophic secretion to sustain neuronal viability[26]. Hypoosteocalcinemia correlates with accelerated cognitive deterioration, underscoring the necessity of osseous-neural axis regulation for systemic health maintenance[2].

Neurotrophic factors, particularly BDNF, are also crucial for cognitive function[233]. BDNF is a key neurotrophic factor, and its expression level is significantly positively correlated with cognitive function. Exercise can promote neuron survival, dendrite development and sudden strengthening by up-regulating BDNF, thereby improving learning and memory abilities[29]. BDNF supports neural growth, dendritic development, and synaptic strengthening, which collectively enhance cognitive abilities[234]. Exercise, especially moderate to high-intensity activity, significantly increases serum BDNF levels, providing evidence for its role in cognitive enhancement[235]. Sustained multimodal exercise regimens demonstrate anti-inflammatory efficacy through suppression of IL-6 and TNF-α production, correlating with cognitive enhancement[236]. Persistent neuroinflammation constitutes a principal etiological factor in neurodegenerative pathogenesis and cognitive degradation[237]. Cerebrospinal inflammatory biomarker elevation exhibits strong associations with neuronal damage and neural stem cell proliferation inhibition^[238,239]^. Exercise-mediated IL-10 induction counteracts inflammatory cascades, establishing mechanistic links between anti-inflammatory modulation and cognitive preservation[240]. Through dual modulation of pro-/anti-inflammatory cytokine balance and pathway activation, physical intervention strategies confer neuroprotective benefits with potential cognitive decline retardation[241].

The bone–brain axis is a crucial pathway in maintaining cognitive function, involving the interaction of bone-derived factors, neurotrophic factors, and other molecules (Table 3)[41]. Exercise enhances the secretion of osteocalcin and ucOCN, which directly influence neuroplasticity and memory, improving cognitive function[50]. Moreover, bone-derived factors modulate microglial activity and reduce neuroinflammation, further protecting the nervous system[6]. Ongoing investigation into exercise-cognition interactions promises development of precision interventions accounting for age-specific and pathological variables, potentially revolutionizing geriatric cognitive preservation and quality-of-life enhancement paradigms.

### Effects of physical activity on neurodegenerative diseases

Neurodegenerative pathologies, particularly AD and PD, constitute major healthcare challenges in aging societies[242]. Exercise has garnered attention for its potential in preventing and treating these diseases, with growing evidence suggesting it can improve cognitive function and provide neuroprotection, possibly through the bone–brain axis, slowing disease progression[243].

Exercise influences brain health by modulating skeletal function, particularly in neurodegenerative diseases (Table 3)[130]. Bone marrow-derived factors, such as OCN and ucOCN, cross the BBB and positively impact brain function[26]. These factors are not only involved in bone metabolism but also affect neuroplasticity, learning, and memory[2]. In animal models of AD and PD, MSCs from bone marrow have shown promise in improving cognitive function and alleviating neurodegenerative changes[244]. Irisin released during physical activity activates hippocampal TrkB/BDNF signaling cascades, stimulating neural stem cell proliferation and differentiation to ameliorate spatial memory deficits in AD murine models^[117]^. Exercise-mediated suppression of IGF1R sumoylation attenuates neuroinflammatory responses in APP/PS1 transgenic mice[29].Bone marrow-derived microglial analogs demonstrate therapeutic potential by reducing β-amyloid deposition and decelerating cognitive deterioration in AD preclinical studies[245]. Preconditioning with bone marrow stromal cells exhibits dopaminergic neuroprotection in PD models, suggesting exercise-enhanced skeletal factor secretion may reinforce neural resilience and plasticity[246]. Osteocalcin and ucOCN, key bone-derived factors, play a critical role in brain function[19]. Osteocalcin enhances synaptic plasticity by promoting neurotrophic factor production and regulating hippocampus-dependent memory through the Wnt-β-catenin pathway[26]. Exercise stimulates osteocalcin secretion, supporting cognitive recovery in neurodegenerative diseases[3]. Furthermore, exercise modulates neurotrophic factors, which further enhances its neuroprotective effects[247]. For instance, exercise can regulate microglial activity, increase anti-inflammatory cytokines (e.g., IL-6, IL-10), and inhibit neuroinflammation, a critical mechanism for protecting the nervous system[141]. In AD patients, reducing neuroinflammation may slow disease progression and improve quality of life[248]. Exercise also shows significant neuroprotective effects in AD[243]. AD-related cognitive dysfunction correlates with amyloid plaque accumulation and tau protein pathology[249]. Physical intervention not only enhances cognitive performance but also modulates cerebral metabolic pathways to retard neurodegenerative progression, offering complementary therapeutic approaches[250]. Recent studies demonstrate that exercise-induced reduction of IGF1R sumoylation significantly attenuates neuroinflammation in AD models, highlighting a molecular link between metabolic regulation and neuroprotection[29]. Studies show that exercise enhances neuroplasticity and synaptic function, aiding cognitive restoration[140]. Transplantation of bone marrow-derived MSCs in animal models improves cognitive deficits and reduces neurodegenerative damage[244]. Osteocalcin, crossing the BBB, helps protect neurons, promoting memory and learning[26]. As exercise increases bone-derived factor secretion, it promotes neural repair and functional recovery[135].

In PD, exercise provides neuroprotective effects by protecting dopaminergic neurons[251]. Pre-treatment with bone marrow stromal cells mitigates dopaminergic neurodegeneration in Parkinson’s mouse models[252]. Studies also show that bone marrow-derived microglia-like cells reduce neuroinflammation and mitigate neurodegeneration, suggesting exercise may be a therapeutic strategy for PD[241]. Exercise modulates the bone–brain axis to mitigate neuroinflammation and improve cerebrovascular function through synergistic interactions between bone-derived hormones and anti-inflammatory cytokines. Osteocalcin binds to GPR158 receptors on microglia, enhancing IL-10 signaling via STAT3 activation. This suppresses NF-κB-driven pro-inflammatory cytokine release. Patients with neurodegenerative diseases, particularly AD and PD, are at higher risk for osteoporosis and fractures, which may be linked to disruptions in the bone–brain axis[142]. Studies suggest that impairments in this axis not only affect skeletal health but also compromise brain function[111]. Therefore, improving the function of bone-derived factors could offer new therapeutic approaches for these diseases[253]. In conclusion, exercise plays a vital role in preventing and treating neurodegenerative diseases by enhancing neuroplasticity and regulating bone-derived factors. The bone–brain axis offers a novel explanation for exercise’s impact on brain health, with osteocalcin and ucOCN serving as potential therapeutic targets. Exercise not only improves cognitive function but may also delay neurodegenerative disease progression, providing additional treatment options for patients.

### Exercise on mood regulation and antidepressant effects

Physical activity enhances cognitive performance and potentially decelerates neurodegenerative pathology progression, offering supplementary therapeutic alternatives for clinical management. As a non-pharmacological intervention, exercise demonstrates significant efficacy in mood enhancement, depressive symptom mitigation, and psychological state regulation[254]. Contemporary research elucidates that exercise counteracts depression through both direct neurophysiological mechanisms and multi-modal support via bone–brain axis modulation, neuroendocrine system regulation, and neurotrophic factor potentiation (Fig. 4)[255].

**Figure 5.**
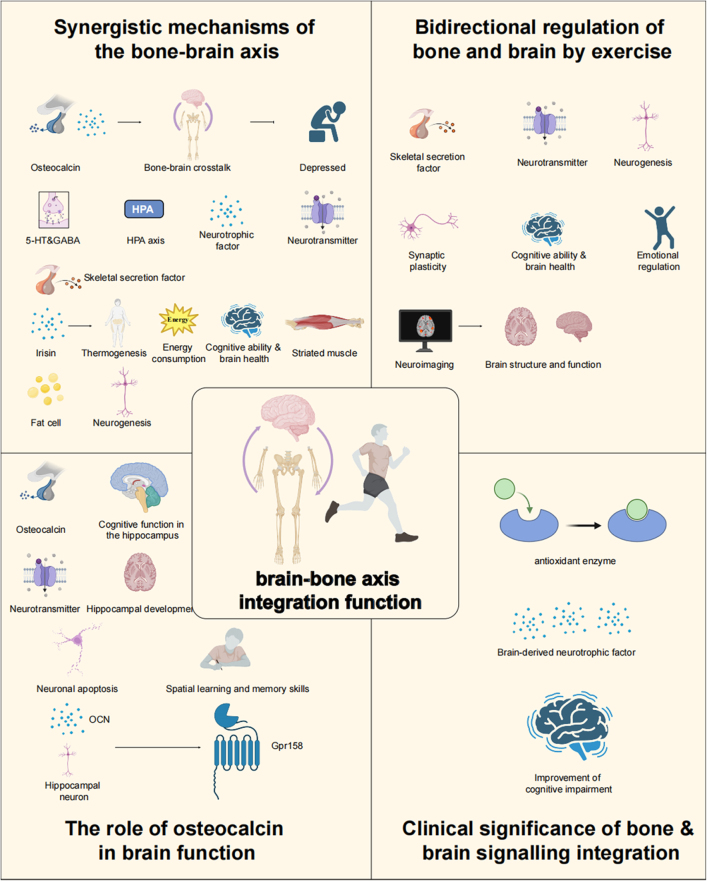
Synergistic mechanisms and clinical implications of the bone–brain axis in regulating brain function and health.

The bone–brain axis serves as a pivotal regulator of affective states[42]. Osseous-derived mediators including osteocalcin (OC) and undercarboxylated osteocalcin (ucOCN) traverse the BBB to influence neural circuitry, thereby modulating mood and cognitive functions[2]. Exercise-induced osteocalcin elevation correlates with emotional stability, while osteocalcin deficiency associates with anxiety-depression comorbidity[256]. The ucOCN isoform exhibits antidepressant properties through neurotransmitter synthesis modulation, neuroplasticity augmentation, and anti-inflammatory action[26]. Physical training stimulates ucOCN secretion, enhancing dopaminergic and serotonergic neurotransmission to alleviate depressive manifestations[257]. The ucOCN isoform exhibits antidepressant properties through neurotransmitter synthesis modulation, neuroplasticity augmentation, and anti-inflammatory action[28]. Exercise further regulates neuroendocrine homeostasis via hypothalamic-pituitary-adrenal (HPA) axis normalization[258]. Chronic HPA axis hyperactivity induces hypercortisolemia, a cardinal biomarker of depressive disorders[259]. Exercise effectively reduces cortisol concentrations, ameliorating stress-induced depressive phenotypes (Fig. 5)[260]. BDNF upregulation through exercise facilitates neuronal regeneration and synaptic repair, constituting a critical antidepressant mechanism[261]. Aerobic protocols significantly elevate BDNF levels, improving depression-related neural network functionality[262].

This figure summarizes the integrative and bidirectional functions of the bone–brain axis in promoting cognitive health, emotional regulation, and brain structure through exercise-induced mechanisms. In the context of synergistic mechanisms of the bone–brain axis, exercise stimulates the secretion of bone-derived factors such as osteocalcin, which crosses the BBB and modulates neurotransmitter systems (e.g., 5-HT and GABA) and the hypothalamic-pituitary-adrenal (HPA) axis, alleviating depression and improving neuroplasticity. Muscle-derived factors such as irisin contribute to fat cell browning, thermogenesis, and energy metabolism, further supporting neurogenesis and cognitive health. The bidirectional regulation of bone and brain by exercise is illustrated through exercise-enhanced skeletal secretion of bone-derived factors that impact neurotransmitter release, neurogenesis, and synaptic plasticity. These processes improve emotional regulation, cognitive ability, brain structure, and functional outcomes, as demonstrated through neuroimaging studies. In the role of osteocalcin in brain function, osteocalcin directly influences hippocampal neuron activity via its receptor Gpr158, reducing neuronal apoptosis and enhancing spatial learning and memory abilities. This underscores the importance of osteocalcin in hippocampal development and brain-wide cognitive processes. The clinical significance of bone–brain signaling integration emphasizes the protective effects of exercise-induced antioxidant enzyme activation and brain-derived neurotrophic factor (BDNF) secretion, which mitigate cognitive impairment by improving neuronal health and oxidative stress balance. These pathways demonstrate the potential clinical utility of targeting the bone–brain axis in neurodegenerative diseases and cognitive disorders. This figure highlights the central role of the bone–brain axis in systemic health, providing critical insights into the therapeutic potential of exercise for cognitive function and neuroprotection.

Neuroinflammatory cascades characterized by IL-6 and TNF-α elevation represent core depression pathomechanisms[263]. Exercise attenuates neuroinflammation through pro-inflammatory cytokine reduction and anti-inflammatory mediator induction (e.g., IL-10)[141]. Aerobic and resistance exercises improve microglial function, helping clear harmful substances from the brain and reducing its inflammatory burden, yielding positive effects on depression treatment (Fig. 4)[264]. Social psychological stress impacts mood health, and in conditions like multiple sclerosis (MS), stress may disrupt bone metabolism and affect mood and cognition through the bone–brain axis[265]. Exercise mitigates negative emotions caused by social stress by modulating stress responses. It enhances an individual’s ability to cope with stress and improves physiological responses, reducing depressive symptoms induced by chronic or traumatic stress[266]. Clinical studies confirm that exercise significantly alleviates depressive symptoms, often yielding results comparable to antidepressant medications[267].Physical activity, whether implemented as monotherapy or adjunctive to pharmacologic interventions, enhances affective states, life quality, and self-management capacity in individuals with mild-to-moderate depressive disorders[268]. This intervention concurrently improves somatic health parameters and social adaptation while mitigating social isolation perceptions[269]. In summary, exercise plays a crucial role in mood regulation and antidepressant effects by influencing the bone–brain axis, neuroendocrine regulation, and neuroinflammation (Table 3). These effects improve mood, alleviate depressive symptoms, and promote overall psychological and physical health, making exercise an effective, holistic approach to managing depression.

### Exercise and its impact on cerebrovascular health

Exercise has become a key focus in research on cerebrovascular health, offering benefits through mechanisms like the bone–brain axis[270]. It not only improves cardiovascular health and bone density but also impacts cerebral blood vessels and the microvascular network, promoting vascular health and slowing cerebrovascular decline and neurodegenerative diseases[271]. The bone–brain axis, a bidirectional regulatory network connecting osseous and neural systems, exerts substantial influence on cerebrovascular homeostasis[2]. Osseocrine mediators like osteocalcin permeate the BBB to modulate cerebral vascular dynamic[26]. Osteocalcin optimizes cerebral microcirculation, enhances hemodynamic perfusion, and reinforces BBB structural integrity[26]. Exercise activates this axis, potentially slowing age-related cerebrovascular decline. Aerobic exercise and moderate physical activity improve cardiovascular health, including heart efficiency, blood pressure regulation, and lipid metabolism, which in turn enhances cerebral blood flow[272]. By improving cardiac health and circulation, exercise ensures that the brain receives adequate oxygen and nutrients, crucial for cerebrovascular function. It also reduces inflammation in the vascular endothelium, improving vasodilation and reducing atherosclerosis risk, all of which support healthy brain blood vessels[273]. Exercise also reduces inflammation in the vascular endothelium, improving vasodilation and reducing atherosclerosis risk, all of which support healthy brain blood vessels[29]. Studies have shown that exercise provides a precise therapeutic target for the improvement of cerebral vascular health and myocardial fibrosis by inhibiting Th17 cell differentiation and regulating the ROS/PI3K/Akt/mTOR axis^[274,275]^.

Exercise-induced biomarkers, such as Irisin and Growth Hormone (GH), also contribute to cerebrovascular health[276]. Exercise-induced irisin fosters cerebrovascular well-being through metabolic optimization, cardiovascular enhancement, hemodynamic regulation, and neuroplastic adaptation[277]. Growth hormone (GH) stimulates hepatic insulin-like growth factor-1 (IGF-1) production, which augments cerebral perfusion, promotes angiogenesis, and supports neural network functionality, collectively sustaining cerebrovascular integrity[278]. For brain metastasis patients, exercise may improve cerebrovascular health indirectly by enhancing cardiovascular and bone health[279]. Research shows that exercise can improve survival and delay brain metastasis progression by optimizing the bone–brain axis, promoting cerebral blood flow, and enhancing bone density in mice Lewis lung carcinoma models[279]. Exercise also directly enhances cerebral blood flow and the brain’s microvascular network. It increases cerebral blood flow, improving oxygen and nutrient delivery to the brain, thereby benefiting brain function[280]. As exercise intensity rises, endothelial function improves, microvascular expansion occurs, and blood flow increases, important for preventing and improving cerebrovascular diseases such as stroke and dementia[271]. Long-term exercise can increase microvascular density, promoting endothelial cell proliferation and angiogenic factor secretion, thus improving blood supply to the brain[281]. Chronic exercise induces microglial phenotypic transition from pro-inflammatory (M1) to anti-inflammatory (M2) states through IL-10 mediated pathways. This immunomodulatory shift downregulates inflammatory biomarkers on microglia, establishing an anti-neuroinflammatory milieu that potentially reduces risks of neurodegeneration and cerebrovascular pathologies including cerebral infarction and microangiopathy. For the elderly, moderate exercise slows cerebrovascular deterioration and improves cognitive functions related to aging[282].

Long-term regular exercise provides sustained protection for cerebrovascular health. It improves cardiovascular function, enhances vascular elasticity, regulates lipid metabolism, and reduces the incidence of conditions like diabetes and hypertension, all contributing to better cerebrovascular health[283]. It also modulates blood lipid levels, reduces vascular inflammation, and helps prevent neurodegenerative diseases linked to cerebrovascular decline, such as AD and PD[271]. In summary, exercise positively impacts cerebrovascular health through both indirect benefits (improving cardiovascular health and bone density) and direct effects (activating the bone–brain axis and promoting angiogenesis)(Table 3). The protective effects on cerebrovascular health are as important as those on brain function. By optimizing skeletal integrity, modulating metabolic homeostasis, and stimulating growth factor activation, physical activity reduces cerebrovascular pathology and neurodegenerative disease susceptibility. This non-pharmacological intervention should constitute a cornerstone in cerebrovascular disease prevention and management, particularly for geriatric populations and chronic disease cohorts.

## Integrative function of the brain–brain axis: connecting the key roles of exercise, bone, and brain

### The definition and overall framework of the bone–brain axis

The bone–brain axis represents a bidirectional communication framework between osseous and neural systems mediated through endocrine, immunological, neural, and metabolic pathways, forming an essential physiological network for organismal health maintenance (Fig. 5)[18]. Beyond its biomechanical role, the skeletal system synthesizes osseous mediators including osteocalcin and irisin that regulate cerebral functions encompassing cognitive processing, affective modulation, and neural preservation[2]. Reciprocally, neural and endocrine feedback mechanisms orchestrate osseous metabolism, establishing dynamic skeletal-neural regulatory circuitry[42].

Physical activity serves as the principal activator of bone–brain axis functionality. Exercise elevates circulating levels of osseous mediators like osteocalcin and irisin, which traverse the BBB to modulate neuronal activity, myocyte function, and adipocyte biology[3]. These mediators govern BDNF expression, neuroinflammatory responses, and metabolic regulation. For example, irisin promotes thermogenesis in adipose tissue, boosts metabolism, alleviates neuroinflammation, and enhances neurogenesis, improving cognitive function[284]. Osteocalcin stimulates neurotransmitter secretion (e.g., dopamine, serotonin) and activates the HPA axis, improving mood and cognition[285].

The bone–brain axis regulates neuroimmune, neuroendocrine, metabolic functions, and neuroplasticity[42]. Bone-derived factors help prevent neurodegenerative diseases by reducing neuroinflammation and improving immune function[286]. The exercise-activated HPA axis balances mood and cognition[287]. Metabolically, osteocalcin and irisin enhance energy expenditure in adipose tissue and muscle metabolism, supporting overall metabolic balance and optimizing brain energy use^[178,288]^. In terms of neuroplasticity, these factors promote neurogenesis, enhancing the brain’s ability to adapt to changes[289]. In conclusion, the bone–brain axis provides a biological basis for the health benefits of exercise and highlights the close interaction between bones and the brain. Through this dynamic interaction, exercise enhances cognitive abilities, improves mood, boosts stress resilience, and reduces disease progression related to neuroinflammation, optimizing overall health. Research into this axis not only deepens our understanding of the skeleton-brain relationship but also informs the application of exercise interventions in both brain and bone health.

### The synergistic mechanism of the bone–brain axis

The bone–brain axis integrates multiple biological pathways to support both skeletal and brain functions, contributing to overall health[42]. The skeletal system influences neural networks through osteocalcin and irisin signaling, while cerebral outputs regulate osseous turnover via neuroendocrine pathways[19]. Exercise functions as the critical modulator of this inter-system crosstalk, amplifying skeletal-neural communication and facilitating functional synergy[89].

A crucial aspect of this synergy is the neuroimmune interaction. Bone-derived factors reduce neuroinflammation and improve the brain’s immune environment, protecting neurons and enhancing cognitive function^[2,19]^. For example, irisin mitigates neuroinflammation and promotes thermogenesis in adipose tissue, providing the brain with energy[284]. Osteocalcin influences microglial activity, supporting neurorepair[290]. This neuroimmune regulation is vital for brain health and may aid in preventing neurodegenerative diseases. In neuroendocrine regulation, bone-derived factors activate the HPA axis, affecting mood, stress responses, and cognitive abilities[197]. Osteocalcin, released during exercise, influences neurotransmitters like serotonin and dopamine, improving mood and memory[26]. Irisin, crossing the BBB, regulates brain metabolism and enhances the brain’s adaptation to stimuli[35]. Together, these factors form a network that optimizes brain and skeletal functions. Metabolic regulation is another key element of the synergistic mechanism. Osteocalcin and irisin enhance energy expenditure in adipose tissue and muscle, maintaining metabolic balance and optimizing brain energy use[178]. Irisin enhances neural functionality through thermogenic activation and metabolic regulation, while osteocalcin optimizes cerebral glucose and lipid utilization to potentiate cognitive performance[288]. Exercise intensifies these metabolic adaptations, conferring dual benefits to skeletal and neurological systems.

Exercise intensifies these metabolic adaptations, conferring dual benefits to skeletal and neurological systems.[291]. Osteocalcin specifically augments hippocampal-dependent learning and memory consolidation, whereas irisin facilitates hippocampal neuronal proliferation and differentiation^[26,284]^. This enhancement of neuroplasticity helps the brain adapt to external changes and slows cognitive decline, especially in aging[292]. Through the synergistic mechanisms of the bone–brain axis, exercise integrates skeletal and brain functions. Bone-derived factors influence the brain via endocrine, immune, and metabolic pathways, improving cognition and emotional well-being. Simultaneously, the brain supports bone health through feedback mechanisms. This bidirectional regulation provides a foundation for using exercise in disease prevention, neurodegenerative disease treatment, and health optimization. The bone–brain axis exemplifies the complex interactions between the skeleton and brain, offering future research prospects for exploring the broad effects of exercise on health.

### Bidirectional regulation of the bone–brain axis by exercise

Exercise exerts a bidirectional regulatory effect on the bone–brain axis, enhancing brain function through skeletal health and vice versa, promoting skeletal metabolism through neural health[76]. It activates various physiological mechanisms that foster the interaction between the skeleton and brain, ultimately enhancing their synergistic functions and improving overall health[293].

Physical activity stimulates osteocalcin and irisin release, which not only enhance osseous mineralization and density but also cross the BBB to optimize neuronal bioenergetics and functional capacity (Fig. 5)^[3,294]^. Osteocalcin enhances neuroplasticity, memory, learning, and emotional regulation, while irisin helps regulate brain energy balance by affecting adipose and skeletal metabolism, supporting cognitive and emotional function^[284,295]^. Thus, exercise boosts both skeletal health and brain function by improving the brain’s energy supply. In turn, brain activity and endocrine changes regulate skeletal health[22]. During exercise, neural-endocrine responses activate hormones (e.g., growth hormone, adrenaline), influencing bone metabolism and structure[150]. Neural regulation via the HPA axis coordinates osteogenic proliferation, differentiation, and mineralization processes[296]. Exercise-induced BDNF elevation promotes bone marrow stromal cell osteogenic differentiation, accelerating skeletal formation and repair[297].

Exercise establishes a bidirectional regulatory mechanism between the brain and bones through neuroimmune modulation[298]. The nervous system releases neurotransmitters (e.g., norepinephrine, serotonin) during exercise, influencing bone metabolism[299]. Norepinephrine stimulates osteoblast proliferation, promoting bone formation[300]. Exercise also reduces inflammation, optimizing the bone immune environment, which further aids bone repair[298]. At the same time, bone-derived factors reduce neuroinflammation, protecting the brain from chronic damage and improving brain function[42]. This neuroimmune regulation not only optimizes bone–brain synergy but also supports exercise interventions for neurodegenerative diseases and osteoporosis[6]. Exercise’s regulatory role is also significant in metabolism. Osteocalcin, irisin, and other factors influence systemic metabolism, promoting energy expenditure and fat metabolism, benefiting both bone health and providing energy for the brain^[301,302]^. Exercise increases skeletal energy consumption, enhancing glucose and fatty acid metabolism, which supports brain metabolic function[303]. Bone-derived factors ensure the brain receives adequate energy during exercise, regardless of intensity[285]. Moreover, exercise enhances neuroplasticity through the bone–brain axis. Exercise-induced bone-derived factors increase neurotrophic factors like BDNF, promoting neuronal growth, synapse formation, and improved memory function[304]. Osteocalcin protects hippocampal neurons, promoting neuroplasticity and cognitive function, while irisin enhances neuronal metabolism through the adipose-nervous system interaction^[26,284]^. This neuroplasticity enhancement enables the brain to adapt to environmental changes and slows cognitive decline associated with aging[305]. In sum, exercise regulates the bone–brain axis through bone-derived factor secretion, neuroimmune modulation, metabolic optimization, and neuroplasticity enhancement. This improves skeletal health, bone density, cognitive function, emotional regulation, and neural repair. Exercise is a potent intervention with broad applications for preventing and treating neurodegenerative diseases, osteoporosis, and other health conditions, offering a new approach to maintaining overall health.

### The relationship between bone-derived factors and brain health

Bone-derived factors, critical regulators of bone metabolism, have gained increasing attention in neuroscience research for their profound effects on both bone and brain health. These factors, including osteocalcin, osteonectin, and osteopontin, influence brain function, neural development, and cognitive preservation through mechanisms such as neuroendocrine signaling, metabolism, and immune modulation[19].

Osteocalcin, a prominent bone-derived factor, connects the skeleton to the brain, circulating through the bloodstream and directly influencing brain function[26]. BDNF binding to cerebral receptors stimulates neurite outgrowth and synaptic remodeling, particularly within hippocampal circuits, thereby enhancing mnemonic and cognitive functions[219]. Osteocalcin is also linked to cognitive function and may have protective effects against neurodegenerative diseases like AD[50]. Deficiencies in osteocalcin are associated with cognitive decline and mental disorders, including depression[306]. Similarly, osteonectin and osteopontin play vital roles in brain health, supporting neural cell development, repair, and neuroplasticity[19]. Osteonectin promotes neuronal repair and has potential therapeutic effects in brain injury and neurodegeneration, while osteopontin aids in neuronal migration and axonal growth, supporting recovery after stroke[307]. Bone-derived factors regulate brain health via multiple mechanisms. They influence the neuroendocrine system, which governs hormones crucial for neural activity. Osteocalcin exerts regulatory effects on the HPA axis, mediating stress response modulation and neuroendocrine activity that governs affective states and behavioral patterns[308]. This skeletal-derived factor further coordinates hormonal systems including insulin and adrenaline signaling, critically influencing metabolic homeostasis and cognitive processes[302]. Circulating osteocalcin levels demonstrate strong correlations with glycemic control and energy equilibrium—physiological prerequisites for optimal neural function and cognitive performance[309]. Skeletal mediators additionally regulate neuroimmune interactions, governing cerebral immune surveillance through microglial activation attenuation and immunocyte functional modulation, thereby providing neuroprotection against AD and PD pathologies^[19,310]^. Chronic neuroinflammatory states characteristic of neurodegeneration are counteracted through osteocalcin-mediated promotion of neural repair mechanism[6].

Bone-derived factors significantly impact cerebral bioenergetics, with osteocalcin serving as a master regulator of osseous mineralization, adipogenesis, and glucose metabolism to maintain systemic metabolic balance[311]. Disruptions in metabolism can lead to cognitive decline, but bone-derived factors optimize metabolic processes, enhancing the brain’s energy supply and supporting cognitive and emotional health[19]. Altogether, bone-derived factors are essential for maintaining brain health. These mediators enhance neurogenesis, synaptic remodeling, and endocrine-immune-metabolic crosstalk, unveiling novel therapeutic targets for neurodegenerative disease management. Expanding research anticipates pivotal clinical applications of skeletal-neural axis modulation in cerebral health preservation strategies.

### The relationship between bone mineral density and cognitive function

The correlation between bone mineral density (BMD) and cognitive performance has attracted growing research interest. Scientific investigations reveal that reduced BMD impacts both skeletal integrity and cerebral health, with particular vulnerability observed in geriatric cohorts^[305]^. Postmenopausal women exhibit accelerated BMD decline due to estrogen deficiency, a process strongly linked to cognitive deterioration[312]. Clinically, diminished BMD elevates risks for cognitive dysfunction and neurodegenerative pathologies, notably AD[313].

#### Association between bone mineral density and cognitive decline

Longitudinal analyses demonstrate a dose-dependent relationship between BMD reduction and cognitive decline velocity in postmenopausal populations. A cohort study monitoring 946 osteoporotic patients identified AD incidence in approximately 38% of cases, underscoring the osteoporosis-cognition nexus[314]. Additionally, due to hormonal fluctuations (such as estrogen, IGF-1, and osteocalcin) in postmenopausal women, the decline in BMD is more pronounced[315]. These hormonal changes not only affect bone health but also influence brain function through the BBB. Studies suggest that these bone-derived factors may regulate cognitive functions in the nervous system by interacting with specific receptors in the brain, thereby impacting cognitive abilities[50].

#### Bone mineral density and Alzheimer’s disease

Alzheimer’s disease patients frequently demonstrate reduced bone mineral density (BMD), particularly those with concurrent osteoporosis. Research confirms that AD patients exhibit marked BMD reduction and elevated hip fracture incidence, further implicating skeletal deterioration in cognitive decline pathophysiology[316].Accelerated femoral bone loss in postmenopausal women correlates strongly with heightened cognitive impairment risk, underscoring the osteoporosis-neurodegeneration nexus[317]. This association may stem from dysregulated osseocrine signaling, where diminished osteocalcin bioavailability exacerbates neural dysfunction through disrupted neuromodulation[50].

#### Bone-derived factors and cognitive function

Osteocalcin (OCN), an important marker of bone remodeling, is significantly reduced in patients with osteoporosis and is also found to be reduced in neurodegenerative diseases such as AD. Studies suggest that osteocalcin not only serves as an indicator of bone health but may also directly impact brain cognitive function[50]. In animal experiments, osteocalcin-deficient mice exhibited cognitive impairments and a reduction in brain monoamine neurotransmitter levels, further confirming the critical role of osteocalcin in regulating cognitive function[219]. Osteocalcin regulates hippocampus-dependent memory by binding to the Gpr158 receptor, which in turn influences learning and memory (Fig. 5). Osteocalcin emerges as a dual-function mediator, orchestrating both skeletal homeostasis and cognitive preservation via neuroregulatory mechanisms[218]. Clinically, hypoosteocalcinemia in elderly females associates with impaired executive function and episodic memory deficits, providing robust evidence for its role in cognitive deterioration.

#### Osteoprotegerin (OPG) and cognitive function

Osteoprotegerin (OPG), secreted by osteoblasts, inhibits bone resorption by binding to RANKL and regulates immune responses. Increasing evidence suggests that OPG plays a crucial role in the nervous system, particularly in the repair of neuronal injuries[318]. OPG promotes neuronal regeneration by modulating immune responses and enhances synaptic plasticity, thereby improving cognitive function. Studies indicate that changes in OPG levels are closely related to the occurrence of neurodegenerative diseases and the progression of cognitive impairments, further underscoring the profound impact of bone metabolism factors on cognitive health[19].

#### The bone–brain axis and cognitive function

The bone–brain axis, a bidirectional signaling network bridging skeletal and neural systems, serves as the principal conduit linking BMD to cerebral function[41].Osseous mediators like osteocalcin and undercarboxylated osteocalcin (ucOCN) traverse the BBB to modulate neuronal activity. BMD depletion alters secretory profiles of these factors, potentially accelerating neurodegeneration and cognitive dysfunction[2].

#### The dual effects of exercise on bone density and cognitive function

As a non-pharmacological therapeutic approach, physical exercise demonstrates dual efficacy in augmenting BMD and optimizing cognitive performance. Scientific investigations confirm that structured physical regimens not only improve osseous integrity but also modulate secretory profiles of skeletal-derived mediators, thereby enhancing neural functionality[319]. Exercise enhances bone metabolism, increases bone mineral density, and improves cerebral blood flow and neural function, which can help reduce cognitive decline. Through its effect on the bone–brain axis, exercise not only improves skeletal health but also significantly slows the decline in cognitive function, especially in the elderly population, thus offering significant preventive benefits[320]. In summary, there exists a close bidirectional relationship between BMD and cognitive function. The role of bone-derived factors provides a biological mechanism to support this relationship. By intervening in BMD, particularly through exercise and other means, cognitive decline may be delayed to some extent, with important clinical implications for the elderly population.

### Exercise optimizes brain function via the bone–brain axis

Physical activity demonstrates recognized therapeutic benefits for cerebral health, with emerging research elucidating its capacity to optimize neural function through skeletal-neural axis modulation^[3,321]^. This reciprocal osseous-cerebral interaction coordinates multiple physiological pathways that enhance neuroprotection, cognitive enhancement, and neurodegeneration prevention[2]. Exercise modulates neural functionality through multifaceted skeletal-neural axis interactions. A principal pathway involves exercise-induced elevation of osseocrine mediators like osteocalcin, which demonstrate neuroactive properties during physical exertion[322]. Osteocalcin enters the brain through the bloodstream, promoting neuronal growth, synaptic plasticity, and neuronal survival, which helps mitigate age- or disease-related neuronal damage[219]. Thus, exercise enhances brain adaptability and plasticity by modulating skeletal activity[323]. In addition to this, exercise improves brain metabolism by enhancing cardiovascular and respiratory function, which boosts blood circulation and oxygen supply—both vital for brain energy metabolism[280]. Exercise stimulates bone and muscle tissues, leading to the release of factors that optimize the brain’s ability to utilize glucose and oxygen, thereby supporting cognitive function. In individuals with chronic diseases or aging-related decline, these metabolic improvements help slow cognitive deterioration and the progression of neurodegenerative diseases^[250,282]^. Exercise also positively impacts the neuroimmune system by reducing neuroinflammation[141]. During exercise, bone-derived factors interact with immune cells in the brain, promoting the release of anti-inflammatory molecules that inhibit chronic inflammation[324]. This is particularly important in mitigating excessive microglial activation, which is linked to neurodegenerative diseases, and helps protect brain health in both older individuals and younger populations, including athletes[241].

Exercise modality parameters (intensity, duration, frequency) critically determine skeletal-neural axis modulation efficacy. Aerobic protocols including brisk ambulation, endurance running, and cycling demonstrate superior capacity for elevating osseous mediator concentrations and cognitive optimization[325]. High-intensity interval training (HIIT) exhibits additional potential for improving cerebral metabolism, mnemonic consolidation, and affective regulation[326]. However, excessive intense exercise can be detrimental, leading to overstrain on both the skeleton and brain, which may result in injury and fatigue[327]. Therefore, moderate, consistent exercise is the optimal approach for maximizing the brain benefits of the bone–brain axis[229]. Collectively, exercise promotes brain function not only by benefiting bone health but also by optimizing brain metabolism, neuroplasticity, and immune responses. Osseous-derived factors constitute central regulators of cognitive processes, emotional homeostasis, and global neurological integrity. Advancing bone–brain axis research positions physical activity as a viable non-pharmacological strategy for cerebral health preservation and neurodegenerative disease mitigation.

### Clinical significance and applications of the brain–brain axis

Osseous-derived mediators serve pivotal functions in cognitive regulation, affective stability, and holistic neurological integrity. Expanding investigations into the skeletal-neural axis position physical activity as a viable non-pharmacological modality for cerebral health preservation and neurodegeneration prophylaxis. The brain–brain axis paradigm elucidates profound interconnections between skeletal and neural homeostasis, revealing novel therapeutic avenues for disease prevention and management[119]. This framework enables clinical strategies targeting osseous health optimization to enhance neurological outcomes, particularly in neurodegenerative pathologies, cognitive dysfunction, and mood spectrum disorders. Within AD and PD research, the skeletal-neural axis assumes critical importance given the pathogenic triad of neuroinflammation, oxidative stress, and synaptic plasticity impairment. Osteocalcin and related osseocrine factors demonstrate neurorestorative potential through synaptic adaptability enhancement, neuronal cytoprotection, and inflammatory cascade attenuation, thereby decelerating cerebral degenerative processes[328]. Combining exercise, diet, and pharmacological interventions to target the bidirectional regulation between bones and the brain offers a promising approach for treating these diseases^[1,41]^.

The skeletal-neural axis introduces novel paradigms for cognitive preservation, particularly in aging demographics. Scientific evidence establishes interconnectivity between osseous integrity and cognitive performance, with osteocalcin and related mediators enhancing cerebral metabolism and synaptic adaptability[2]. Proactive interventions combining moderate physical activity with calcium/vitamin D supplementation demonstrate dual efficacy in skeletal fortification and cognitive optimization, offering integrated preventive strategies in geriatric care[232]. Within affective disorder management, this axis shows therapeutic relevance for depression and anxiety through neuroendocrine equilibrium restoration[42]. These conditions correlate with disrupted neuroplasticity and inflammatory cascades[329]. Exercise-induced osteocalcin secretion modulates dopaminergic and serotonergic neurotransmission, providing mechanistic links between osseous health and emotional regulation[306].Combined exercise and bone density management emerge as viable adjunct therapies for mood disorders, as illustrated in Figure 5[330].

Furthermore, osseous homeostasis critically influences neurorehabilitation outcomes post-central nervous system trauma (e.g., stroke, spinal cord injury) by supporting neural repair mechanisms in plasticity-compromised environments[331]. Enhancing bone health and regulating bone-derived factors could accelerate neural repair and improve neurological recovery[332]. Exercise has already shown promise in post-stroke recovery, and further research on the relationship between exercise and bone-derived factors may open new avenues for treating central nervous system injuries. Finally, the brain–brain axis has implications for treating osteoporosis, which is not only a risk factor for fractures but also associated with cognitive decline and mood disorders[111]. Promoting bone health and slowing osteoporosis progression can reduce fractures and improve brain health[50]. In osteoporosis patients, interventions like exercise and nutritional supplementation can improve both bone and brain health. The brain–brain axis also provides a new perspective on preventing and intervening in cognitive impairments, particularly in the aging population. Studies show a correlation between bone health and cognitive function, with bone-derived factors like osteocalcin enhancing brain metabolism and neuroplasticity[2]. Interventions that promote bone health, such as moderate exercise and supplementation with calcium and vitamin D, can improve cognitive function and delay decline in older individuals, presenting an integrated strategy for clinical prevention[232]. In the realm of emotional and mental health, the brain–brain axis has clinical value for mood disorders like depression and anxiety[42]. These conditions are linked to neuroendocrine imbalances, neuroplasticity dysfunction, and inflammation[329]. Exercise, which improves bone health, promotes the secretion of bone-derived factors like osteocalcin, which in turn regulate neurotransmitters such as dopamine and serotonin, influencing mood and behavior[306]. Combining exercise with bone health management offers a potential adjunctive strategy for treating mood disorders (Fig. 5)[330]. Bone health also plays a critical role in the recovery following central nervous system injuries, such as stroke and spinal cord injury, where neuroplasticity and repair capacity are often diminished[331].

## Summary of clinical studies and experimental data

### Clinical and animal experimental data on the intervention of exercise in neurological diseases

**Figure 6.**
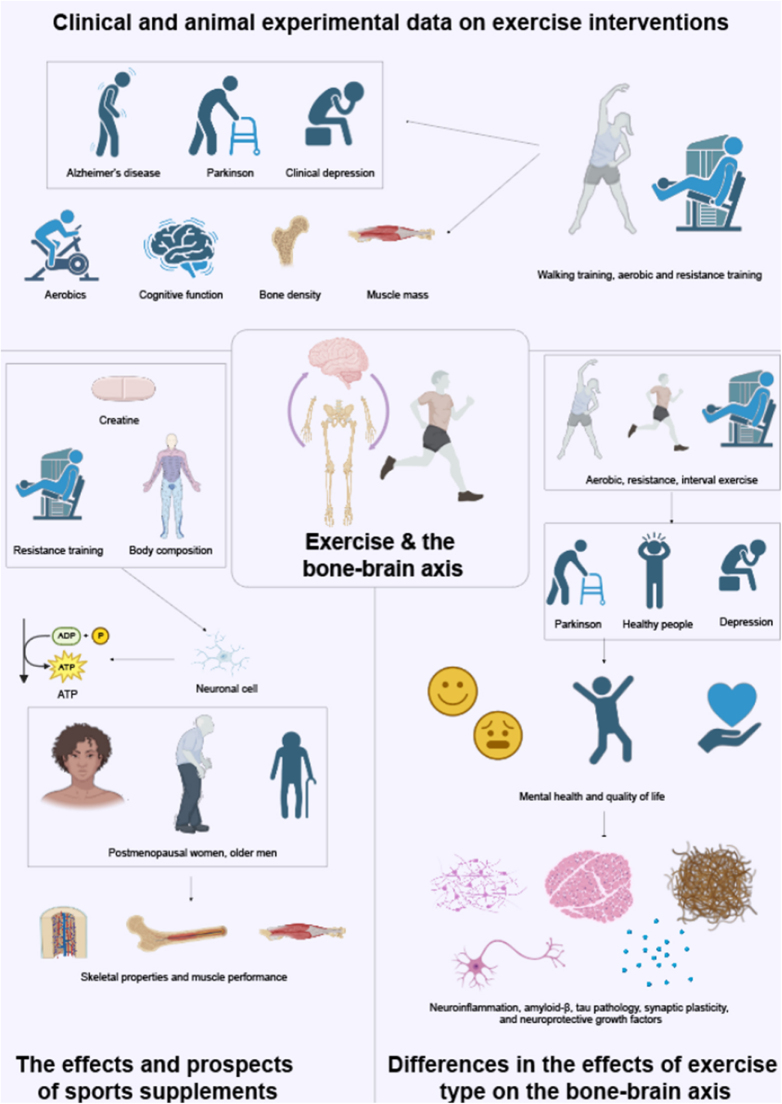
The impact of exercise interventions on the bone–brain axis: insights from clinical and animal experimental studies.

Exercise has been extensively shown to benefit neurological diseases through clinical studies and animal experiments, particularly in treating AD, depression, and attention deficit disorder (Fig. 6, Table 4)^[333,334]^. Both animal models and human trials demonstrate significant improvements in cognitive function, emotional state, and brain health[335].

This figure provides an overview of the effects of various exercise interventions on the bone–brain axis, as demonstrated through clinical and animal studies. Exercise modalities such as walking, aerobic, resistance, and interval training are highlighted for their roles in improving cognitive function, bone density, and muscle mass, particularly in populations affected by neurodegenerative diseases (e.g., Alzheimer’s disease and Parkinson’s disease), clinical depression, and aging-related decline. The central panel illustrates the systemic impact of exercise on the bone–brain axis, emphasizing its influence on skeletal properties, neuronal cell health, and muscle performance. Aerobic and resistance training contribute to better body composition and increased energy metabolism (ATP production), particularly in postmenopausal women and older men, where the benefits extend to maintaining skeletal health and muscle function. On the right, differences in exercise types are shown to modulate neuroinflammation, reduce amyloid-β accumulation, enhance synaptic plasticity, and promote the release of neurotrophic growth factors, leading to improvements in mental health and quality of life. Aerobic and interval exercises are noted for their positive effects on emotional regulation and cognitive resilience. Additionally, the lower panel explores the potential of sports supplements, such as creatine, in synergizing with exercise interventions to optimize bone–brain axis function. These interventions collectively emphasize the importance of tailored exercise programs and nutritional strategies for preventing and managing neurodegenerative diseases, improving quality of life, and promoting healthy aging. This comprehensive representation underscores the therapeutic potential of exercise and its interaction with the bone–brain axis across diverse populations and conditions.

**Table 4:** Effects of different types of exercise on the bone–brain axis

	Neurological health	Bone strength	Muscle mass	Mental Health
Aerobic exercise	Increases glucose uptake and energy metabolism in the brain	-	-	Reduced depression scores and improved attention deficit symptoms
Resistance Exercise	Enhances the adaptability of the nervous system and reduces neuroinflammation	Increase bone load and strengthen bone density	Increase muscle strength and endurance	Significantly improves adults’ emotional state and reduces symptoms of depression
Intermittent exercise	Promote the secretion of neuroprotective factors and improve cerebral blood flow	-	Reduce fat content	-
Yoga	Regulates the self-repair mechanism of the nervous system	-	Improve body flexibility	Effectively relieves anxiety and depression symptoms

In animal models of neurodegenerative diseases, especially AD and PD, aerobic exercise (PE) promotes neurogenesis and cognitive function[243]. These effects are mirrored in clinical studies, where exercise, particularly aerobic activity, has clear benefits. For example, elderly individuals who regularly engage in aerobic exercise show improvements in bone density, muscle mass, and cognitive function[336]. An MRI study found that 6 months of aerobic exercise (three times a week, 60 minutes per session) increased gray and white matter volumes in the anterior cingulate cortex, correlating with improved cognition[337]. Resistance training also improves cognitive function and physical health[338]. A meta-analysis by Lv *et al* showed that exercise interventions significantly improved cognitive function in attention deficit disorder patients (ADHD), with resistance training being particularly effective[339]. In a systematic review of 5 studies (93 patients), it was found that 72.4% of ADHD patients showed cognitive improvements following resistance training (*P*<0.05). This suggests resistance training is highly effective in enhancing cognitive function[340].

Physical activity exerts beneficial effects on cerebral energy metabolism. Sustained aerobic training enhances cerebral glucose utilization, thereby supporting cognitive homeostasis[341]. For individuals with mild attention-deficit disorders, structured moderate-to-vigorous aerobic protocols (e.g., treadmill/bicycle ergometer training: 60-minute sessions, thrice weekly over 16 weeks) demonstrate measurable cognitive enhancement[342]. Additionally, exercise reduces neuroinflammation, which is crucial for protecting against neurodegenerative damage[141]. In the elderly, exercise not only improves cognition but also prevents and delays attention deficit disorder onset[282]. A study showed that sedentary elderly individuals had a 53% higher incidence of attention deficit disorder compared to those who exercised, highlighting the preventive role of exercise in neurodegenerative diseases[342]. Resistance training similarly confers somatic health benefits[343]. Ahn *et al* documented significant improvements in muscular strength, endurance, and cardiovascular parameters following 5-month elastic band resistance interventions in attention-deficit cohorts[344]. Collectively, exercise serves as a cornerstone intervention for neurological disorders through cognitive optimization, neurometabolic activation, neuroinflammatory suppression, and neurogenic stimulation. Aerobic and resistance modalities demonstrate particular efficacy, with long-term moderate-to-high intensity regimens synergistically improving neural and physical health parameters, establishing their critical role in neurodegenerative disease prevention and therapeutic strategies.

### Support for the bone–brain axis function by exercise supplements

Exercise supplements, such as creatine and vitamin D, have gained increasing attention for their role in enhancing exercise performance and supporting the bone–brain axis[345]. These supplements not only improve training effectiveness but also positively affect muscle mass, energy metabolism, neural function, and skeletal health (Fig. 6)[346].

Creatine, a key energy supplement, is widely used to improve high-intensity and endurance training performance[347]. It supports energy resynthesis in muscles, the brain, and bones through the phosphocreatine system, enhancing ATP production, maintaining ion gradients, and supporting neurotransmitter release and synaptic function[348]. Studies have shown that creatine supplementation improves cognitive function, especially in conditions like stress or hypoxia, where it alleviates mental fatigue and boosts cognitive performance[349].

For the elderly, creatine supplementation improves muscle strength and body composition, especially when combined with resistance training[350]. High doses (e.g., 20 grams per day for 7 days) significantly increase muscle strength and exercise capacity, while lower doses (e.g., 1 gram per day) have weaker effects[351]. Long-term low-dose supplementation (e.g., 3 grams per day for 2 years) has shown little impact on muscle growth or performance[351]. The standard dosing regimen for creatine supplementation typically involves a loading phase of 20 grams per day (divided into 4 doses) for 5-7 days, followed by a maintenance dose of 3-5 grams per day. However, the potential of creatine to enhance brain function, particularly through increased brain creatine levels, is still being explored[352]. Future studies may explore higher doses for more significant effects on the bone–brain axis.

Vitamin D supplementation is also crucial for bone–brain axis function[353]. It regulates calcium and phosphorus metabolism, increases bone density, and supports bone strength[354]. Vitamin D deficiency is linked to cognitive decline and neurodegenerative diseases[355]. Adequate vitamin D levels improve brain energy metabolism and neuroprotection, especially in aging and neurodegenerative conditions[356]. Beyond bone health, vitamin D regulates neuroinflammation and promotes neural cell growth and repair, supporting overall bone–brain axis health[357].The established nutritional guidelines for vitamin D specify age-dependent requirements, with older adults typically requiring 800-1000 IU (20-25 mg) daily to sustain optimal 25-hydroxyvitamin D concentrations. Concurrent administration of vitamin D with structured aerobic and resistance training demonstrates synergistic enhancement of osseous density and cognitive metrics in geriatric populations. In conclusion, creatine and vitamin D are important for supporting the bone–brain axis. Creatine enhances energy supply and cognitive function, while vitamin D promotes bone health and neuroprotection. These supplements show promise in improving physical health, enhancing cognitive function, and providing neuroprotection, making them valuable for overall bone–brain axis function.

### Implications for surgical and clinical interventions

Emerging insights into exercise-mediated bone–brain axis modulation hold profound clinical implications, particularly for surgical rehabilitation and cognitive preservation in high-risk groups such as elderly or osteoporotic patients. Patients undergoing orthopedic surgeries, such as hip and knee replacements or fracture repairs, often experience prolonged periods of immobility, which can lead to both physical deconditioning and cognitive decline[358]. Implementing structured exercise regimens that incorporate weight-bearing activities and balance training can facilitate recovery. Post-fracture rehabilitation protocols incorporating progressive resistance regimens not only accelerate osseous regeneration but also elevate cognitive performance through neurotrophic mediator release (e.g., BDNF)^[359,360]^. Evidence suggests that incorporating weight-bearing and resistance exercises shortly after fracture repair can significantly improve both bone density and cognitive outcomes[361]. A structured program that begins with guided walking and progresses to more complex strength training can aid in restoring both physical and cognitive functions. Research indicates that patients recovering from spinal surgery benefit from early physical therapy that includes flexibility and strength training exercises. These integrated interventions optimize physical recuperation while fortifying neural resilience via neuroprotective factor upregulation[362]. Combining NMES with traditional rehabilitation strategies can promote rehabilitation to enhance muscle activation and improve mobility, ultimately leading to improved cognitive and functional outcomes[363]. In conclusion, a rehabilitation program that incorporates physical exercises, cognitive tasks, and NMES may enhance both neuroplasticity and muscle recovery, addressing the dual concerns of physical mobility and cognitive health in surgical patients.

**Table 5:** Recommendations for exercise prescriptions for clinical populations.

Clinical target	Exercise modality	Intensity	Duration	Frequency
Osteoporosis prevention	Resistance training	70-85% 1 RM	30-60 minutes	2-3 times/week
	Aerobic exercise	Moderate (50-70% HRR)	150 minutes/week	5 times/week
	High-impact activities	Moderate to high	Varies	2-3 times/week
Mild cognitive impairment	Aerobic exercise	Moderate to high	30-60 minutes	3-5 times/week
	HIIT	75-90% VO_2_ max	20-30 minutes	2-3 times/week
Postmenopausal women	Resistance training	70-85% 1 RM	30-60 minutes	2-3 times/week
	Aerobic exercise	Moderate (50-70% HRR)	150 minutes/week	5 times/week
	Combined modalities	Varies	30-60 minutes	3-5 times/week

### The effects of different types of exercise on the bone–brain axis

Physical activity enhances systemic health through multifaceted interactions with the bone–brain axis, with modality-specific benefits demonstrated across exercise types. Empirical studies confirm that aerobic conditioning, resistance protocols, and interval training collectively improve cognitive performance, mitigate neurodegenerative symptomatology, and optimize skeletal integrity[232]. Each modality differentially modulates bone–brain axis components, particularly influencing neural vitality, osseous resilience, musculoskeletal mass, and psychological well-being[364].

Aerobic training demonstrates recognized efficacy in neural optimization, particularly for cognitive enhancement and affective regulation (Table 4)[365].This modality elevates cerebral perfusion and stimulates neurotrophin production (e.g., BDNF), thereby augmenting synaptic plasticity and cognitive capacity[366]. Systematic engagement in moderate-to-vigorous aerobic protocols (e.g., running, aquatic exercises, cycling) at 3-4 weekly sessions exhibits longitudinal antidepressant effects and cognitive improvement[367]. Mechanistically, aerobic exercise enhances cerebral glucose utilization and bioenergetic efficiency, particularly beneficial in Alzheimer’s pathology[368]. A six-month aerobic intervention in elderly cohorts increased gray/white matter volumes in the anterior cingulate cortex with concomitant cognitive enhancement[221]. Such training also attenuates attention-related cognitive deterioration[369].

Resistance exercise exerts positive neuromusculoskeletal effects through bone–brain axis modulation. By augmenting muscular strength and endurance, this modality improves both somatic and neural functionality[16]. Resistance training enhances neural adaptability, suppresses neuroinflammatory cascades, and optimizes neuronal metabolism[88]. Regular implementation (weight training, elastic band exercises) significantly elevates mood states, alleviates depressive symptoms, and provides therapeutic benefits in PD and AD management (Fig. 6)[370]. This intervention upregulates neuroprotective mediators (IGF-1) while reducing inflammatory biomarkers (e.g., IL-15), demonstrating clinical utility in ADHD symptom management[371]. Furthermore, resistance protocols increase bone mineral density, decelerate osteoporotic progression, and reduce fracture risk, particularly in geriatric and postmenopausal populations[372].

Intermittent training protocols demonstrate therapeutic potential for neurodegenerative populations, particularly those with mild cognitive impairment and Alzheimer’s pathology[373]. It boosts the body’s metabolic rate, improves cardiovascular function, reduces body fat, and enhances brain function^[361,374]^.Compared to sustained aerobic regimens, interval-based exercise elicits superior metabolic adaptation, enhances neuroprotective mediator secretion, optimizes cerebral perfusion, and elevates cognitive performance[375].

Low-impact regimens such as yogic practices exert measurable effects on skeletal-neural communication networks[376]. Mindfulness-oriented yoga interventions effectively mitigate anxiety-depression comorbidity while enhancing psychological well-being and functional capacity in Parkinson’s cohorts[377]. Compared to traditional stretching or resistance training, yoga enhances neuroprotection through improved flexibility, posture, and mental relaxation[378]. The meditation and breathing techniques in yoga help regulate the nervous system’s self-repair mechanisms, further benefiting brain function[379]. The mechanism is achieved by stimulating the vagus nerve to correct the insufficient activity of the PNS and GABA systems, as well as reducing non steady state loads.

### Differential effects of exercise intensity, frequency, and mode on the bone–brain axis

The intensity, frequency, and mode of exercise significantly influence the bone–brain axis. Research shows that exercise effectiveness is not only determined by the type of exercise but also by its intensity, frequency, and duration[380]. An appropriate balance of these factors is crucial for improving neural health, bone strength, and mood regulation, with different exercise intensities and modes offering distinct benefits for cognitive function, mood, and neurodegenerative disease prevention (Fig. 6)[381].

Exercise intensities ranging from 40-80% of VO_2_ reserve (VO_2_R) or heart rate reserve (HRR) demonstrate optimal efficacy for cognitive enhancement and mental health optimization[255]. Studies have found that this intensity reduces depression scores and has lasting positive effects[330]. It stimulates the release of antioxidant enzymes and neurotrophic factors like BDNF, IGF-1, and VEGF, promoting neuroplasticity and cognitive improvements[88]. This intensity is especially beneficial for the elderly and individuals with mild cognitive impairment, as it can delay neurodegenerative changes and improve cognitive function[382]. Although moderate to high-intensity exercise is highly effective, lower-intensity exercise still provides benefits, particularly for mood and anxiety[383]. Light activities like walking or gentle yoga may not significantly improve cognitive function but are valuable for reducing anxiety and depression[384]. These protocols offer enhanced safety and implementation feasibility for individuals with physical comorbidities or compromised baseline fitness.

Exercise frequency is another critical factor. For patients with depression or cognitive impairment, exercising 3-4 times per week is optimal[385]. Research suggests that higher frequency results in greater reductions in depressive symptoms and cognitive improvements[385]. However, exercise plans should be personalized to match individual needs for the best outcomes[386]. The duration of exercise interventions also affects their effectiveness. Short-term exercise (less than 12 weeks) can alleviate depression and improve mood, but its impact on cognitive function is limited. Long-term exercise (over 24 weeks) has more substantial effects, particularly on spatial memory and attention[339]. Prolonged interventions lead to adaptive changes in brain function, significantly improving symptoms in neurodegenerative diseases. The mode of exercise further impacts intervention outcomes. Endurance exercises, such as running and cycling, improve mood, with cycling showing a greater effect on anxiety reduction (Table 4)[387]. Resistance training, such as weightlifting, is particularly effective in improving cognitive function and neuroprotection, especially in regulating neuroinflammation and neurotransmitter release[388]. Yoga and mindfulness practices also benefit the bone–brain axis by reducing mental stress and improving symptoms of anxiety and depression[389].

## Future perspectives

Advancing research on the bone–brain axis is driving the formulation of individualized therapeutic approaches, particularly for neurodegenerative disease management[42]. Exercise interventions are gaining attention for their potential in treating conditions like AD and depression[390]. Scientific evidence confirms exercise-induced modulation of osseous-neural interactions yields measurable physiological enhancements, with adjunct nutritional interventions like creatine and vitamin D supplementation demonstrating synergistic efficacy[346]. Resistance training protocols incorporating creatine supplementation significantly improve muscular performance and anthropometric profiles in geriatric populations[391]. The differential effects of various exercise types on the bone–brain axis provide important insights for developing personalized exercise regimens. In the future, through cohort studies of people with different exercise patterns, we will deeply explore the occurrence and development of diseases and related factors, and provide valuable scientific support for precise prevention[360].

**Figure 7.**
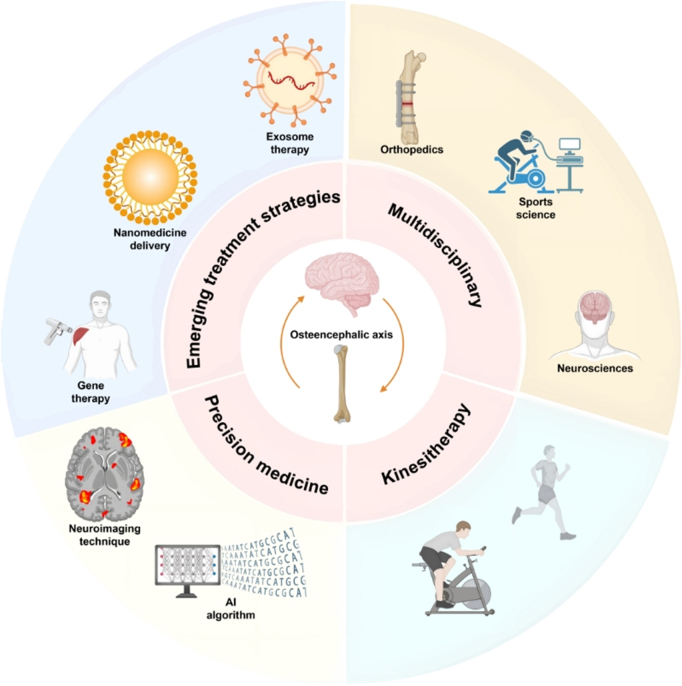
Emerging therapeutic strategies and multidisciplinary approaches centered on the bone–brain axis.

In the realm of precision medicine, personalized exercise prescriptions are becoming increasingly feasible[392]. The integration of artificial intelligence (AI) and neuroimaging technologies promises to advance this field further^[360,393]^.Combined with bioinformatics analysis and a variety of algorithms and tools, it provides technical support for bone–brain axis-related gene expression and pathway analysis^[394,395]^. For example, using single-cell transcriptomics to analyze the dynamic changes of cell subpopulations in the neuroimmune microenvironment can more accurately reveal the interaction mechanism between bone-derived factors and neuroinflammation, providing a new perspective for targeted intervention[71]. AI can analyze large datasets to identify individual physiological characteristics and risk factors, aiding in the customization of exercise plans^[360,396]^. When combined with neuroimaging and machine learning, these technologies can predict disease progression in AD and stroke patients and help create more targeted rehabilitation strategies^[360,397]^. The rise of wearable devices and smart sensors, coupled with AI, will provide real-time monitoring and feedback, improving the precision and effectiveness of clinical interventions (Fig. 7)^[360,398]^.

This figure illustrates the bone–brain axis as a central framework that integrates emerging therapeutic strategies and multidisciplinary approaches to enhance skeletal, neurological, and overall systemic health. The bone–brain axis, which represents the intricate connection between bone and brain, serves as a critical target for novel interventions and advancements in both clinical and research contexts. Surrounding the central axis are four interrelated domains that emphasize innovative approaches to understanding and leveraging this connection. Emerging treatment strategies, including exosome-based therapies, nanomedicine delivery systems, and gene therapies, represent cutting-edge modalities for targeting both bone and brain health. These approaches aim to modulate the molecular and cellular mechanisms underpinning the bone–brain axis, offering potential applications for neurodegenerative diseases, bone disorders, and systemic health conditions. Multidisciplinary approaches, combining orthopedics, sports science, and neuroscience, underscore the necessity of collaborative efforts to address the biomechanical, metabolic, and neurological dimensions of the bone–brain axis. These disciplines work together to advance therapies and interventions that holistically address the interconnected systems. Precision medicine, powered by technologies such as advanced neuroimaging and AI-driven algorithms, provides a personalized approach to understanding individual variations in bone–brain interactions. These tools enable the development of targeted therapeutic strategies that align with specific patient profiles. Kinesiotherapy, incorporating exercise-based interventions like aerobic and resistance training, highlights the role of physical activity in activating pathways within the bone–brain axis. These pathways support neuroplasticity, enhance synaptic growth, and improve both cognitive function and bone density, making exercise a cornerstone of health optimization. At the center of the figure, the bone–brain axis symbolizes the integration of these approaches, reinforcing the bidirectional relationship between bone and brain as a foundation for systemic health. This conceptual framework emphasizes the importance of merging traditional disciplines with emerging technologies to pioneer innovative solutions for disease prevention, rehabilitation, and the improvement of quality of life.

Emerging therapies like gene therapy, exosome therapy, and nanodrug delivery offer new avenues for regulating the bone–brain axis[399]. The noninvasive approach through ultrasonic nasal drops has shown potential for regenerating damaged neurons in CNS injuries[400]. The key challenge is to achieve the most complete transduction of the target structure while avoiding leakage into adjacent areas or the surrounding space of blood vessels. Among them, MRI guided enhanced convective delivery (iMRI CED) is a future focus[401]. Extracellular vesicles (EVs) carrying BDNF enhance neuronal cytoskeletal stability and demonstrate therapeutic efficacy in preclinical PD models^[402,403]^. Advanced brain-targeted nanodelivery systems employ three primary strategies: receptor-mediated targeting (transferrin/LRP/lactoferrin receptors), adsorption-mediated transport (cationic polymers/CPPs), and transporter-facilitated mechanisms (P-gp/GLUT1)[404]. Innovative therapeutic approaches including gene editing, exosome-based delivery, and nanopharmaceuticals enable precise modulation of bone–brain axis dynamics[405]. Network pharmacology integrated with experimental validation provides novel methodologies for exploring natural compounds’ regulatory effects on skeletal-neural interactions, accelerating drug discovery processes^[405,406]^. The crosstalk between muscle-derived stem cells (MDSCs) and periosteal progenitor cells (PPCs) represents a critical frontier in bone–brain axis research[407]. Investigating exercise-mediated modulation of osseocrine signaling may yield breakthrough therapies for neurodegenerative disease prevention and progression management[6]. Imitation smart materials simulate the natural tissue environment to realize osteochondral regeneration, and combine with exercise intervention to optimize the bone repair effect[408]. MXenes, a class of electroactive materials, have been extensively explored for their application in repairing electroactive tissues and organs such as the brain and spinal cord[409]. Integrating bioinformatics technology with single-cell sequencing, cooperation between exercise science, neuroscience and orthopedics will promote the application of bone–brain axonology and deepen our understanding of how exercise affects brain health (Fig. 7)^[31,405^, p5,^410]^. Exercise therapy’s role in treating depression is gaining recognition, particularly as a non-pharmacological self-management tool[411]. It shows significant synergistic effects when combined with medication and psychotherapy[412]. Exercise therapy is cost-effective, easy to implement, and has fewer side effects, improving patients’ quality of life^[255,413]^. Future research should prioritize the clinical application of exercise in mental health, especially for depression. As we better understand the mechanisms of the bone–brain axis, personalized interventions, precision medicine, interdisciplinary collaboration, and emerging therapies will drive progress, opening new pathways for treating cerebrovascular health and neurodegenerative diseases.

## Conclusion

This review synthesizes emerging evidence linking skeletal system signaling to brain function, with a specific focus on the bone–brain axis as a mediator of exercise benefits. By integrating findings from molecular biology, neuroscience, and exercise physiology, we highlight novel endocrine roles of osteokines such as osteocalcin and irisin in promoting cognition and mental health. This interdisciplinary perspective contributes to the growing understanding of exercise as a systemic intervention for neurodegeneration. Future studies should focus on personalized and precise intervention strategies. The combination of creatine supplementation and exercise is recommended as an effective approach for achieving optimal health outcomes, and the safety of creatine has been confirmed[414]. Exercise-induced expression or activation of peroxisome proliferator-activated receptor gamma coactivator 1-alpha (PGC-1α) in skeletal muscle may exert potential antidepressant effects by regulating peripheral metabolism, suppressing inflammatory responses, and promoting the secretion of neuroprotective factors[255]. Furthermore, advances in neuroimaging techniques, non-invasive brain stimulation technologies, and the application of artificial intelligence in interdisciplinary research provide new perspectives for precision medicine and personalized exercise interventions[415]. Future research should particularly focus on elderly populations, exploring the potential of creatine supplementation in gene therapy and nanomedicine delivery, and further investigating how exercise regulates the secretion of factors by osteocytes, thus offering innovative intervention strategies for the prevention or delay of neurodegenerative diseases.

## Data Availability

Data sharing is not applicable to this article as it is a systematic review of existing literature.
